# Ultrasmall Au_10–12_ Nanoclusters: A Novel Platform for ^223^Ra‐Targeted Tumor α‐Therapy

**DOI:** 10.1002/exp2.70144

**Published:** 2026-02-24

**Authors:** Yifei Jiang, Huizhen Yang, Guoping Jia, Wangxi Hai, Rongyi Huang, Peng Wang, Min Zhang, Danni Li, Yizhou Chen, Xiao Li, Biao Li, Chunfu Zhang

**Affiliations:** ^1^ Department of Nuclear Medicine, Ruijin Hospital, School of Biomedical Engineering Shanghai Jiao Tong University Shanghai China; ^2^ Anhui Provincial Laboratory of Optoelectronic and Magnetism Functional Materials School of Chemistry and Chemical Engineering Anqing Normal University Anqing China; ^3^ Department of Biomedical Engineering School of Engineering China Pharmaceutical University Nanjing China; ^4^ Department of Nuclear Medicine Shanghai Changhai Hospital Shanghai China; ^5^ Department of Nuclear Medicine, Inselspital University of Bern Bern Switzerland; ^6^ Institute of Applied Physics Chinese Academy of Sciences Shanghai China; ^7^ National Engineering Research Center of Advanced Magnetic Resonance Technologies for Diagnosis and Therapy Shanghai China

**Keywords:** ^223^Ra labeling, DFT simulation, gold nanoclusters, PET imaging, targeted alpha therapy

## Abstract

^223^Ra, a potent α‐emitting radionuclide, is currently limited to treating bone metastases in clinical practice. This constraint stems from the lack of stable chelating agents capable of withstanding its high recoil energy and the five decay daughters produced during decay, hindering its application in targeted α‐therapy (TAT). Ultrasmall gold nanoclusters (AuNCs) exhibit favorable pharmacokinetics, featuring tunable blood half‐life, renal clearance, and low hepatic/splenic sequestration. Given gold's high atomic number and structural flexibility, we hypothesized that AuNCs could encapsulate ^223^Ra and its decay progeny, serving as an effective carrier for tumor TAT. Density functional theory simulations revealed that Au_10–12_ nanoclusters, particularly a catenane‐structured Au_10_, could incorporate ^223^Ra and its daughters with favorable energy dynamics. We then engineered α_v_β_3_‐targeted AuNCs co‐labeled with ^223^Ra and ^68^Ga (^68^Ga/^223^Ra@AuNCs‐RGD). Molecular docking confirmed receptor specificity, while in vitro and in vivo studies demonstrated tumor‐specific targeting. PBPK modeling and Monte–Carlo simulations showed prolonged circulation (*t*_1/2β_ = 139.4 min), renal clearance, sustained tumor retention, and efficient tumor energy deposition. The ^68^Ga/^223^Ra@AuNCs‐RGD platform achieved complete tumor regression and stimulated antitumor immunity, suggesting potential for TAT–immunotherapy synergy. This study establishes a theoretical foundation for stably doping ^223^Ra into gold clusters and provides a framework for developing novel ^223^Ra‐based α‐radiopharmaceuticals with significant clinical translation potential.

## Graphical abstract

Encapsulating ^223^Ra in Au_10‐12_ nanoclusters and functionalizing them with RGD peptides enables targeted α‐therapy and immune microenvironment remodeling in non‐osseous tumors like triple‐negative breast cancer. This study establishes a foundational framework for novel ^223^Ra‐labeled α‐radiopharmaceuticals and shows strong potential for clinical application.

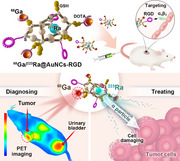

## 1 Introduction

Targeted α‐therapy (TAT) has emerged as a highly effective and targeted approach for cancer treatment, offering distinct advantages over traditional therapies such as external beam radiation therapy (EBRT) and chemotherapy [1]. Unlike β and γ radiation, which have lower linear energy transfer (LET), α particles are high‐LET emitters that deliver dense energy deposition [2, 3]. This results in severe DNA damage, including double‐strand breaks that are irreparable, ultimately inducing apoptosis. This makes TAT highly effective against cancers that have developed resistance to conventional treatments, including β radiation and chemotherapy. Additionally, TAT demonstrates relatively fewer side effects, as its radiation is primarily confined to the tumor site, minimizing damage to normal tissues and reducing systemic toxicity [4, 5]. These unique physical properties position TAT as a promising therapeutic strategy, warranting further research and clinical exploration.

Among the various α‐emitting radionuclides, ^223^Ra stands out due to its ability to emit four α particles during its decay process, inducing clustered DNA damage and apoptosis in tumor cells, particularly in malignant bone tumors and metastases [6]. Recent clinical approval for palliative treatment in hormone‐refractory prostate cancer with bone metastasis has propelled ^223^Ra into the clinical [7, 8]. However, despite these advantages, the application of ^223^Ra is limited by its reliance on bone affinity, making it suitable only for bone‐targeted therapies [9, 10]. The high recoil energy of α particles (100–200 keV) and multi‐decay daughters poses challenges in stable chelation with conventional macrocyclic metal ion complexes, hindering its broader application for tumor therapy. To overcome these limitations, there is an urgent need to develop innovative carrier systems capable of specifically delivering ^223^Ra to tumor tissues, thereby expanding its therapeutic potential [11].

One way to address these limitations is to use nanocarriers, which can incorporate α‐emitting radionuclides and also encapsulate their decay daughters to enhance their labeling stability and tumor accumulation [12, 13]. Moreover, surface modifications with targeting molecules could endow them with tumor specificity [14]. For instance, previous studies have demonstrated ^223^Ra and ^225^Ac could be stably encapsulated in MOF matrices [15], single‐atom nanozymes [16], and polymer micelles [17], respectively. However, the relatively larger size of these nanoparticles has led to significant uptake in the liver, spleen, and bone marrow after intravenous injection, resulting in severe side effects.

Gold nanoclusters (AuNCs), consisting of several to hundreds of gold atoms, have an atomic precision structure and molecule‐like property [18, 19]. AuNCs with ultrasmall sizes (below 5 nm) exhibit favorable pharmacokinetic properties, including renal clearance, low liver and spleen uptake, and tunable blood half‐life, which may provide an ideal carrier for radionuclides to develop novel tumor‐targeted radiopharmaceuticals [20]. AuNCs have been labeled with ^99m^Tc and ^177^Lu for tumor diagnosis and treatment [21]. Previously, we have demonstrated that multiple radioisotopes, including ^68^Ga, ^64^Cu, ^89^Zr, and ^89^Sr, could be efficiently and stably incorporated into AuNCs [22, 23]. Due to its high atomic number, effectively cushioning recoil energy of α particles during decay, we infer that gold nanoclusters may be a robust carrier of ^223^Ra for developing tumor TAT beyond bone metastasis [24, 25].

**FIGURE 1:**
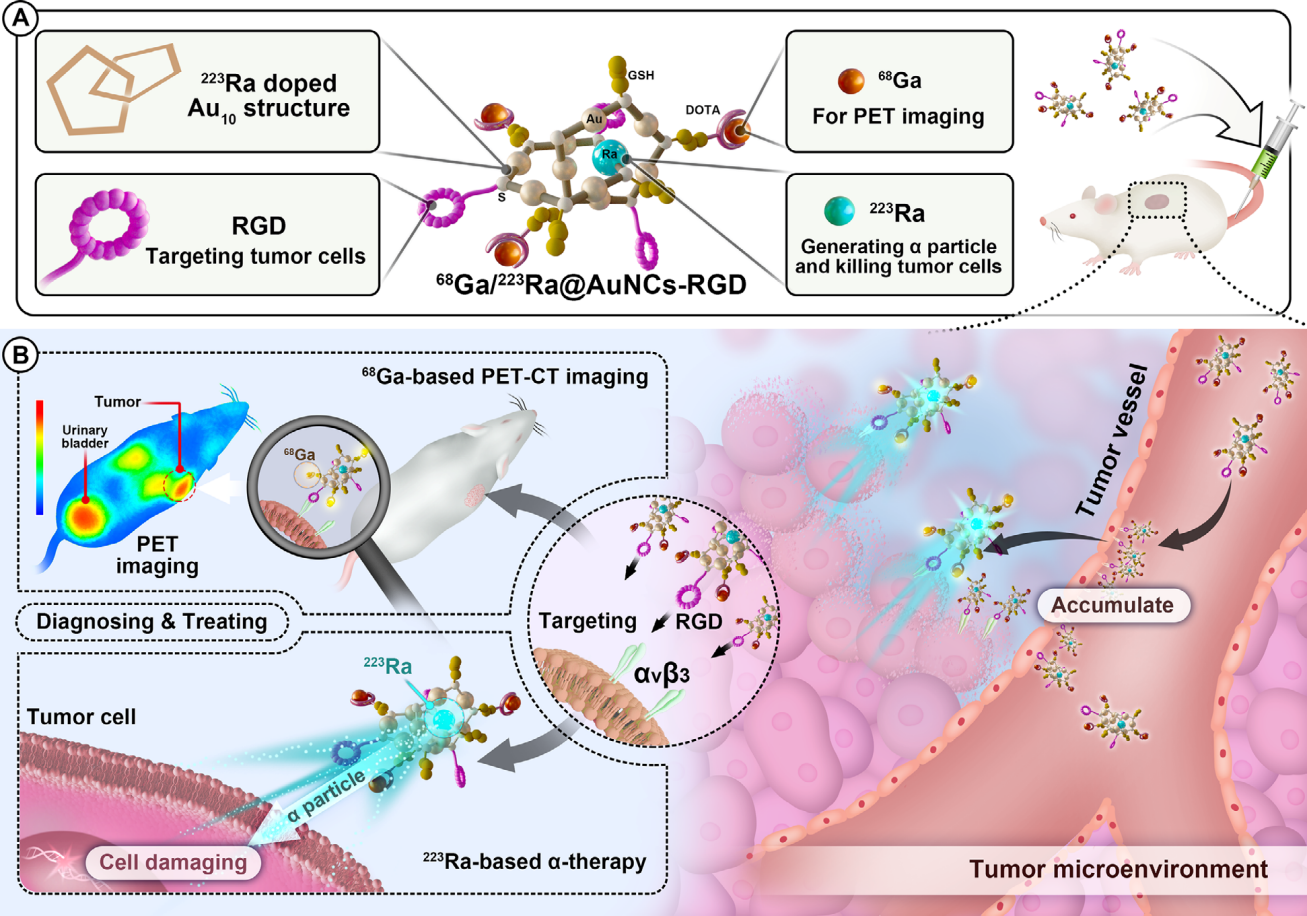
Schematic illustration of radionuclide‐labeled gold nanoclusters for TAT of tumor. (A) Synthetic procedure of ^68^Ga/^223^Ra@AuNCs‐RGD. (B) The mechanism of tumor TAT by ^68^Ga/^223^Ra@AuNCs‐RGD guided by PET imaging.

Therefore, in this study, by doping ^223^Ra into gold nanoclusters and introducing RGD peptide as a model targeting a molecule during synthesis, we developed ^223^Ra‐targeted alpha therapy for non‐bone tumors. The AuNCs consist of 10–12 gold atoms, and we carefully studied ^223^Ra doping mechanism through density functional theory (DFT) simulations. To guide the therapy, we further functionalized the clusters with the macrocyclic chelator DOTA and labeled them with the positron‐emitting radionuclide ^6^ ^8^Ga (^68^Ga/^223^Ra@AuNCs‐RGD). Molecular docking analysis revealed the effective binding of ^68^Ga/^223^Ra@AuNCs‐RGD to the α_v_β_3_ integrin, while in vitro cell uptake experiments and in vivo PET imaging validated ^68^Ga/^223^Ra@AuNCs‐RGD could specifically target tumors. As a result, TAT mediated by ^68^Ga/^223^Ra@AuNCs‐RGD significantly inhibited tumor growth and profoundly reversed the tumor immunosuppressive microenvironment after the therapy (Figure 1). Physiologically based pharmacokinetic (PBPK) modeling and Monte–Carlo simulations revealed favorable pharmacokinetics of the probe, featuring tunable renal clearance, low hepatic/splenic sequestration, prolonged tumor retention, and efficient energy deposition in tumors. This study demonstrates the potential of Au_10–12_ nanoclusters as carriers of ^223^Ra to extend its application to non‐bone tumors, opening new avenues for the development of ^223^Ra α‐radiopharmaceuticals.

## 2 Result and Discussion

### 2.1 Synthesis and Characterization of ^68^Ga/^223^Ra@AuNCs‐RGD

Ligand‐protected gold nanoclusters, with their molecule‐like properties and atomic precision structure, are promising carriers for ^223^Ra labeling to develop target α therapy [26, 27, 28]. For synthesis of ^68^Ga/^223^Ra@AuNCs‐RGD, plain AuNCs were first synthesized through reduction of chloroauric acid by reducing glutathione (GSH). Electrospray Ionization Mass Spectrometry (ESI‐MS) analysis revealed that the main product of the as‐synthesized cluster was Au_10−12_GSH_10−12_ (Figure S1A), which was consistent with the previous report [29]. The cluster was stable in mouse serum with a hydrodynamic size of 1.92 ± 0.15 nm (Figure S2) and a zeta potential of −21.20 ± 0.83 mV.

RGD peptide could specifically bind α_v_β_3_ integrin receptors and has been extensively studied for tumor‐targeted radionuclide therapy (TRT) [30]. To explore whether TRT using AuNCs as a carrier was plausible, c(RGDyC) peptide, as a model molecule and co‐reductant, was introduced during the synthesis. The hydrodynamic size of RGD‐functionalized AuNCs (AuNCs‐RGD) was 2.01 ± 0.13 nm (Figure S2), with predominant structures corresponding to molecular formulas of Au_10_GSH_7_RGD_3_, Au_10_GSH_8_RGD_2_, Au_11_GSH_8_RGD_3_, and Au_12_GSH_8_RGD_4_ (Figure S1B). The zeta potential of AuNCs‐RGD was −11.61 ± 0.59 mV.

**FIGURE 2:**
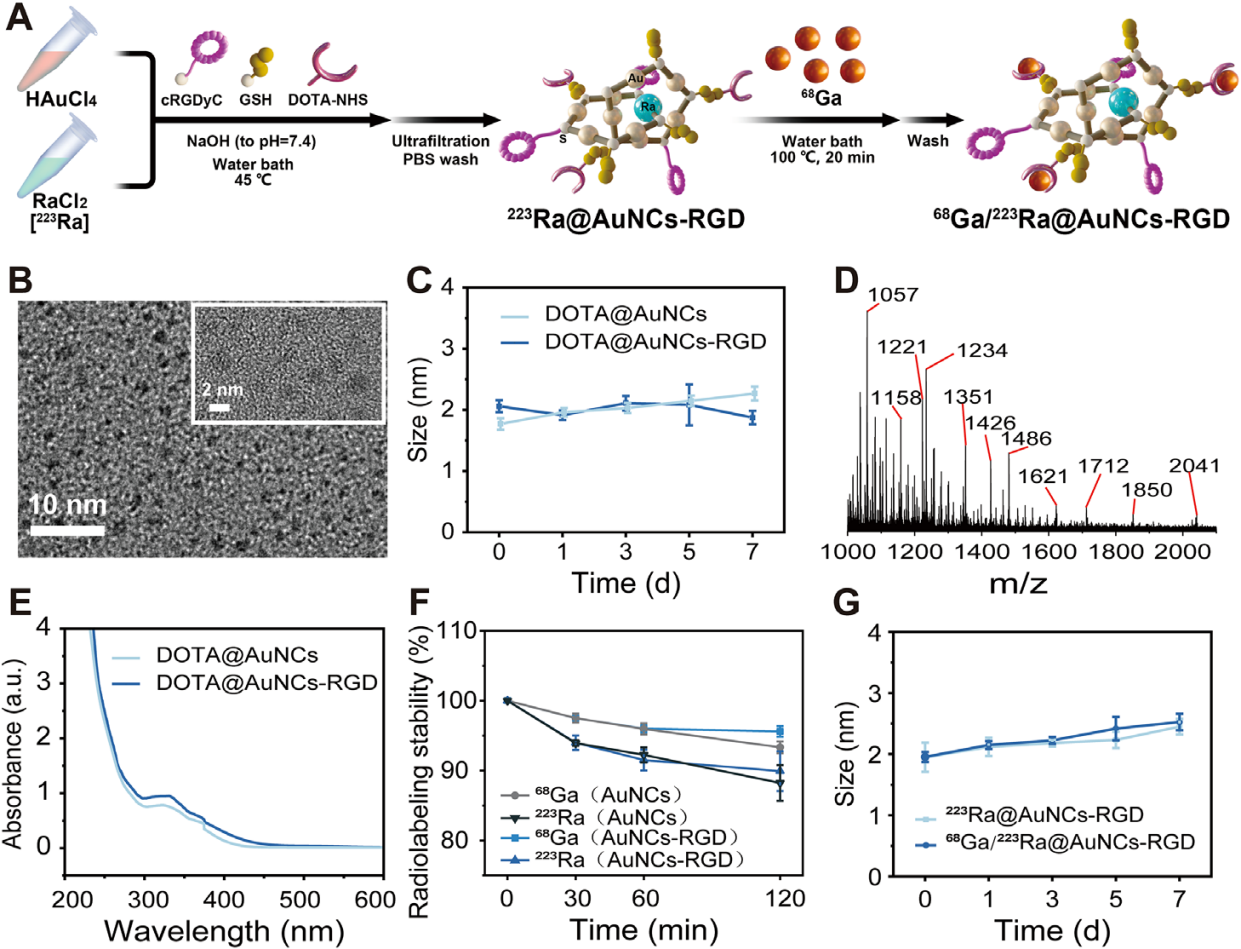
Characterizations of AuNCs. (A) Schematic illustration of the synthesis of the dual radionuclide‐labeled AuNCs (^68^Ga/^223^Ra@AuNCs‐RGD). (B) Representative TEM images of DOTA@AuNCs‐RGD. (C) Colloidal stability of DOTA‐functionalized AuNCs (DOTA@AuNCs and DOTA@AuNCs‐RGD), and the hydrodynamic sizes were determined by DLS. (D) ESI mass spectrum of DOTA@AuNCs‐RGD. (E) UV–vis absorption of DOTA‐functionalized AuNCs (DOTA@AuNCs and DOTA@AuNCs‐RGD). (F) Radiolabeling stability of ^223^Ra and ^68^Ga in mouse serum. (G) Colloidal stability of ^223^Ra and ^68^Ga co‐labeled AuNCs‐RGD (^68^Ga/^223^Ra@AuNCs‐RGD) in mouse serum.

^223^Ra, a therapeutic radionuclide, emits X‐rays (70–90 keV) and γ‐rays (150–300 keV) subsequent to α‐decay [31]. However, the utility of ^223^Ra for SPECT imaging is limited by its low permissible administered dose and the weak intensity of associated γ photons [15]. To achieve clearer therapeutic guidance, DOTA was introduced onto the clusters, allowing ^68^Ga labeling through DOTA‐functionalized GSH (DOTA‐AuNCs). The coupling efficiency of DOTA with GSH was 86.93 ± 2.13%, quantified by the fluorescamine method (Figure S3). Transmission electron microscopy (TEM) analysis further confirmed the ultrasmall size of the DOTA‐AuNCs, with an average size of 1.62 ± 0.11 nm, well below the glomerular filtration threshold of ∼5.5 nm (Figure S4). After being simultaneously functionalized with c(RGDyC) peptide and DOTA (DOTA‐AuNCs‐RGD), DOTA‐AuNCs‐RGD has a uniform morphology with 1.94 ± 0.09 nm observed by TEM (Figure 2B). Both DOTA‐AuNCs and DOTA‐AuNCs‐RGD were stable in physiological condition, and the DLS size was below 3 nm after incubation in mouse serum for 7 days (Figure 2C). Zeta potentials of these two kinds of AuNCs were −26.11 ± 0.61 mV and −14.92 ± 0.39 mV, respectively. The predominant components of DOTA‐AuNCs were Au_10_GSH_6_(GSH‐DOTA)_4_, Au_10_GSH_5_(GSH‐DOTA)_5_, Au_11_GSH_5_(GSH‐DOTA)_6_, and Au_12_GSH_7_(GSH‐DOTA)_5_, while those for both RGD and DOTA functionalized AuNCs were Au_10_GSH_3_(GSH‐DOTA)_4_RGD_3_, Au_11_GSH_3_(GSH‐DOTA)_4_RGD_4_, and Au_12_GSH_4_(GSH‐DOTA)_3_RGD_5_ (Figure 2D and Figure S1C). UV–vis spectra showed distinct peaks at 330 and 375 nm, consistent with the Au_10−12_(SG)_10−12_ reported previously (Figure 2E) [29].

The mechanism for synthesis of AuNCs was through first reduction of Au^3+^ into Au^+^ by the thiol groups in cystine in GSH peptide and simultaneously forming Au(I)‐GSH complexes; these complexes then polymerize into [Au(I)‐GSH]_n_ aggregates, which are subsequently thermalized into AuNCs, as elucidated by Briñas [32]. When c(RGDyC) peptide is introduced, we inferred that its thiol groups in cystine may participate in the reduction and polymerization processes, similar to that in GSH [33]. As a result, c(RGDyC) peptide was immobilized on the clusters after thermolysis. Moreover, the cluster size can be fine‐tuned by adjusting the pH value of the reaction media, with DOTA‐AuNCs‐RGD exhibiting a monodisperse DLS size of 1.93 ± 0.07 nm under physiological conditions (pH 7.4) (Figure S5). This method offers an effective way for the rational design of AuNCs with atomic precision and tumor‐targeting properties, enabling their application in targeted α therapy.

Due to its high decay recoil energy and five radioactive decay daughters, there is no suitable complex for ^223^Ra labeling [34]. Nanoparticles with large sizes have been proposed to trap ^223^Ra inside for labeling; however, high accumulation in the liver and spleen leads to significant radiation toxicity, limiting their in vivo application [35]. We inferred that AuNCs may serve as an effective carrier for ^223^Ra labeling, as the high atomic number (Z) of Au may counteract the recoil energy, while their ultrasmall size facilitates rapid clearance from normal organs, minimizing off‐target radiation exposure [36, 37]. Therefore, we tried to dope ^223^Ra (29 µCi, Xofigo, Bayer Pharma, Germany) into Au_10–12_ nanoclusters via an in situ co‐reduction approach in the presence of GSH and RGD peptide (Figure 2A). The radiolabeling efficiency was 81.42 ± 3.51% with a specific activity of 5.53 µCi mg^−1^, and the radiolabeling was stable, remaining over 85% radioactive on the cluster after 4 days of incubation in mouse serum at 37°C (Figure 2F and Figures S6 and S7).

^223^Ra is a therapeutic radionuclide, emitting four α particles and two β particles from each decay [6]. ^68^Ga was then labeled on the nanocluster surface through DOTA for PET imaging‐guided therapy. The hydrodynamic size of ^223^Ra and ^68^Ga co‐labeled (^68^Ga/^223^Ra@AuNCs‐RGD) AuNCs was 1.96 ± 0.08 nm (Figure S8), without appreciable differences with that of AuNCs‐RGD. The radiolabeling efficiency of ^68^Ga was 76.1% and stable, and there was more than 95% radioactivity retained on the AuNCs after incubation in mouse serum for 120 min at 37°C (Figures S9–S11). In addition, ^223^Ra‐doped (^223^Ra@AuNCs‐RGD) and ^223^Ra and ^68^Ga co‐labeled (^68^Ga/^223^Ra@AuNCs‐RGD) AuNCs were stable in mouse serum, and the hydrodynamic sizes were around 2.5 nm after incubation for 7 days (Figure 2G).

### 2.2 Doping Simulation

**FIGURE 3:**
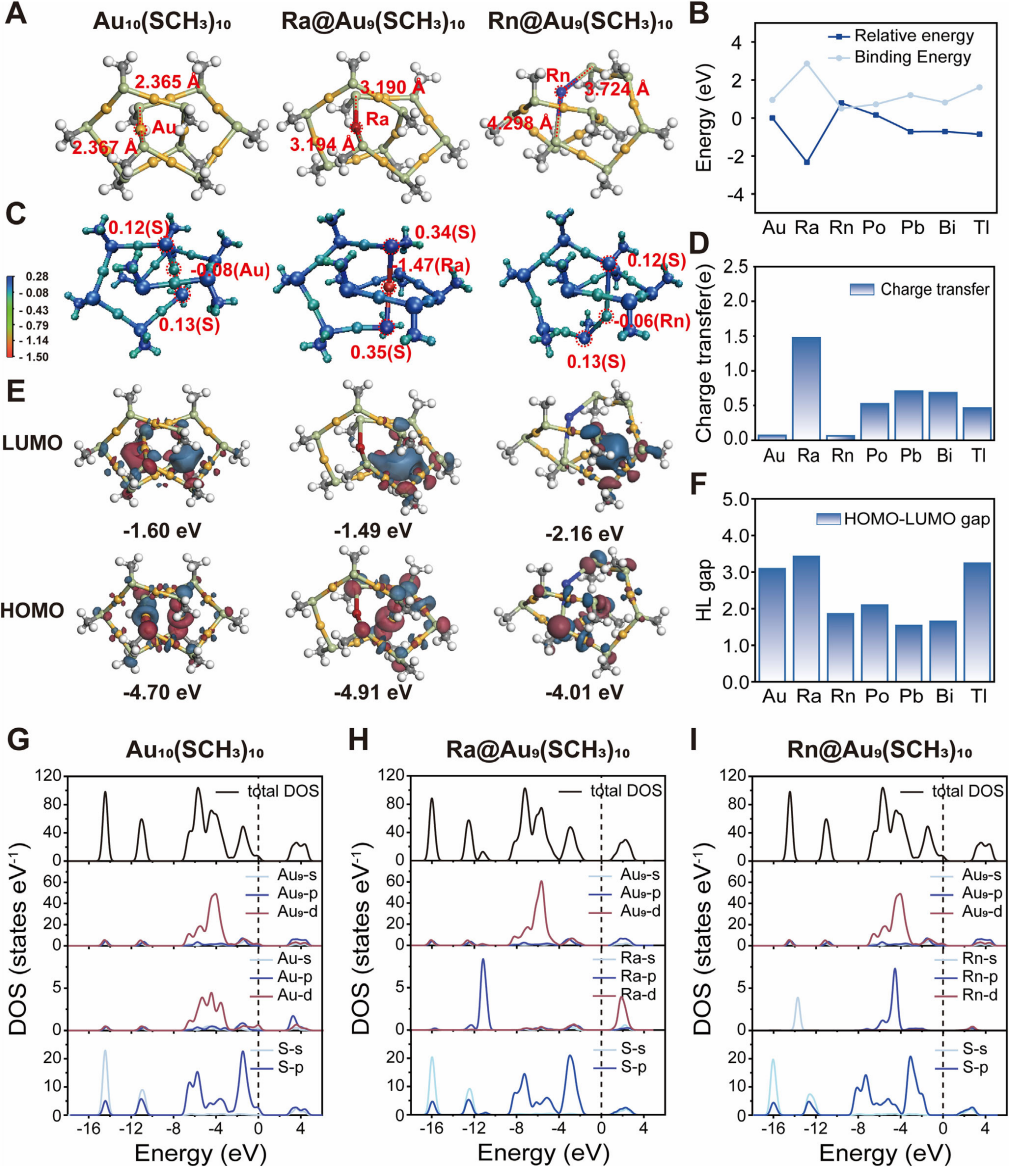
DFT calculations for the ^223^Ra and its progeny radionuclides‐doped Au_10_ clusters. (A) The optimized structure of the heteroatom‐doped Au_10_ clusters displayed by the ball‐and‐stick model and the bond length of the Au, Ra, and Rn atom with the adjacent S atom were annotated. Carbon atoms are in gray, hydrogen atoms in white, sulfur atoms in light green, gold atoms in yellow, radium atoms in red, and radon atoms in blue. (B) Relative energy and binding energy for the heteroatom‐doped Au_10_ clusters. (C) The Bader charge analyses of the heteroatom‐doped Au_10_ clusters. The numerical value indicates charge transfer of Au, Ra, and Rn atoms and adjacent S atoms. (D) The charge transfer of the heteroatom before and after doping. (E, F) HOMO−LUMO gaps for the heteroatom‐doped Au_10_ clusters (F) and their corresponding energy levels (E). (G–I) Total and partial density of states for Au_10_ (G), Ra‐doped (H), and Rn‐doped (I) clusters. The Fermi level is presented as a dashed vertical line and shifted to zero.

^223^Ra decays into multiple progeny radionuclides (^219^Rn, ^215^Po, ^211^Pb, ^211^Bi, ^207^Tl). To reveal the mechanism of ^223^Ra stably doping in Au_10–12_, DFT calculations on the perspective of energetics and electronic structure were performed. The Au_10_ cluster, constituting the largest population among the clusters, served as the initial structure for simulations and as a control. The Ab Initio Random Structure Search (AIRSS) was performed to predict the structures of two model compounds [38], Au_10_(SCH_3_)_10_ and Ra‐doped Au_10_(SCH_3_)_10_ (Ra@Au_9_(SCH_3_)_10_), excluding surface functional groups. The predicted structure of Au_10_(SCH_3_)_10_ was a catenane configuration with two interconnected five‐membered rings (Au_5_(SCH_3_)_5_), aligned with the recent literature reports [39, 40, 41] (Figure 3A and Figure S12). DFT calculations, conducted using the Vienna Ab Initio Simulation Package (VASP), optimized the structures of daughter nuclide‐doped clusters and determined their minimum energy configurations.

We first analyzed energies of heteroatom‐doped Au_10_ clusters (denoted as M@Au_9_, where M represents the heteroatom), with the computationally derived relative energies illustrated in Figure 3B. Among these, Ra@Au_9_ exhibited the lowest relative energy, nearly 2.33 eV lower than that of the pristine Au_10_, while Rn@Au_9_ had the highest one. This discrepancy arises from the significantly longer and unequal Rn–S bond lengths (4.298 and 3.724 Å) compared to the Au–S bond (2.365 and 2.367 Å), leading to local structural deformation around the Rn atom. To assess the stability of ^223^Ra doping in Au_10_ clusters, the formation energy (Δ*E*) was calculated. The negative formation energy of Ra (−1.16 eV) indicates that the doping process is energetically favorable [42], confirming the ease of ^23^Ra incorporation into Au_10_ clusters. Binding energy (*E*_b_) calculations further evaluated the stability of heteroatom‐doped clusters (Figure 3B). Notably, Ra@Au_9_ showed the highest binding energy (2.86 eV), significantly greater than that of the pristine Au_10_ cluster (0.95 eV), highlighting the strong interaction between Ra and the Au_9_ framework. In contrast, Rn@Au_9_ had the lowest binding energy (0.50 eV) due to its longer bond lengths and structural deformation. The binding energies of Po, Pb, Bi, and Tl were similar to that of Au_10_.

To elucidate the high binding energy of Ra@Au_9_, we analyzed the projected crystal orbital Hamilton population (pCOHP) and conducted Bader charge analysis. The stability of heteroatom doping is primarily determined by the strength of the heteroatom–sulfur (M–S) bonds. pCOHP analysis quantified the bonding/anti‐bonding interactions, with the integral of COHP values up to the Fermi level (|IpCOHP|) used to assess bond stability. All M–S bonds exhibited negative COHP values, indicating bonding contributions (Figure S13) [43].

To further investigate the role of electrostatic interactions in doping stability, we analyzed and compared the electronic structures of various heteroatom‐doped Au_10_ clusters using Bader charge analysis (Figure 3C,D and Figure S14). The charge transfer within Ra‐doped nanoclusters may be further enhanced or inhibited due to variations in the electronic structures and electronegativity of the heteroatoms involved during the decay process. This analysis quantified charge redistribution within the clusters, revealing a significant charge transfer (1.47 |e|) for the Ra atom in Ra@Au_9_, consistent with the strong interaction between Ra and the Au_9_ framework [44]. Notably, all daughter nuclides exhibited higher charge transfer than Au, except for Rn, whose behavior was similar to Au – a finding consistent with the binding energy results.

The highest occupied molecular orbital (HOMO)–lowest unoccupied molecular orbital (LUMO) gaps of the heteroatom‐doped clusters were examined to assess their electronic stability. Ra@Au_9_ displayed the largest HOMO–LUMO gap (3.42 eV), further supporting its exceptional stability among the doped clusters [45] (Figure 3E,F and Figure S15). Finally, to obtain a deeper insight into the electronic properties of heteroatom‐doped Au_10_ clusters, we analyze the total and partial density of states (PDOS; Figure 3G–I and Figure S16). The PDOS revealed significant changes in the electronic structure following doping. Specifically, the PDOS exhibited a pronounced leftward shift after doping with different atoms, indicating the introduction of new electronic energy levels by the impurity atoms. In the initial Au_10_ structure, a strong overlap between the 5d orbitals of Au and the 3p orbitals of S was observed, leading to robust orbital hybridization and strong covalent bonding. Additionally, hybridization between the doped atoms and the p and d orbitals of neighboring Au atoms was evident. In the Ra‐doped structure, hybridization between the 5d orbitals of Ra and the 5p orbitals of Au was observed above the Fermi level. In the Tl‐doped structure, new peaks arising from its electronic structure were accompanied by hybridization between the 6p orbitals of Tl and the 5p orbitals of Au above the Fermi level, suggesting enhanced interactions. In contrast, the Rn‐doped structure showed minimal overlap with other atomic orbitals, indicating relatively weak interactions. In summary, our DFT simulations demonstrate that ^223^Ra and its daughter nuclides can be doped into Au_10_ clusters. The high binding energy, significant charge transfer, and large HOMO–LUMO gap of Ra@Au_9_ underscore its exceptional stability within the gold nanocluster framework, providing critical insights for the development of ^223^Ra‐based radiopharmaceuticals.

### 2.3 Molecular Docking and In Vitro Targeting Ability

AuNCs possess unique features, including an atomically precise structure, molecule‐like properties, and a protein‐like hierarchical architecture [19]. To evaluate whether the targeting molecule immobilized on the cluster still keeps specificity to its receptor, a molecular docking analysis of the interaction strength between RGD‐modified AuNCs and the α_v_β_3_ integrin receptor was first performed. Based on mass spectrometry analysis and DFT simulation results, we designed and optimized two nanocluster systems: RGD peptide‐modified nanocluster Au_9_Ra_1_RGD_3_(SCH_3_)_7_ (Ra@AuNCs‐RGD) and plain gold nanocluster Au_9_Ra_1_GSH_3_(SCH_3_)_7_ (Ra@AuNCs). To reduce computational demands, excess glutathione groups were replaced with methyl groups. Both Ra@AuNCs‐RGD and Ra@AuNCs were docked with the α_v_β_3_ integrin receptor, with a standalone RGD peptide serving as the control.

**FIGURE 4:**
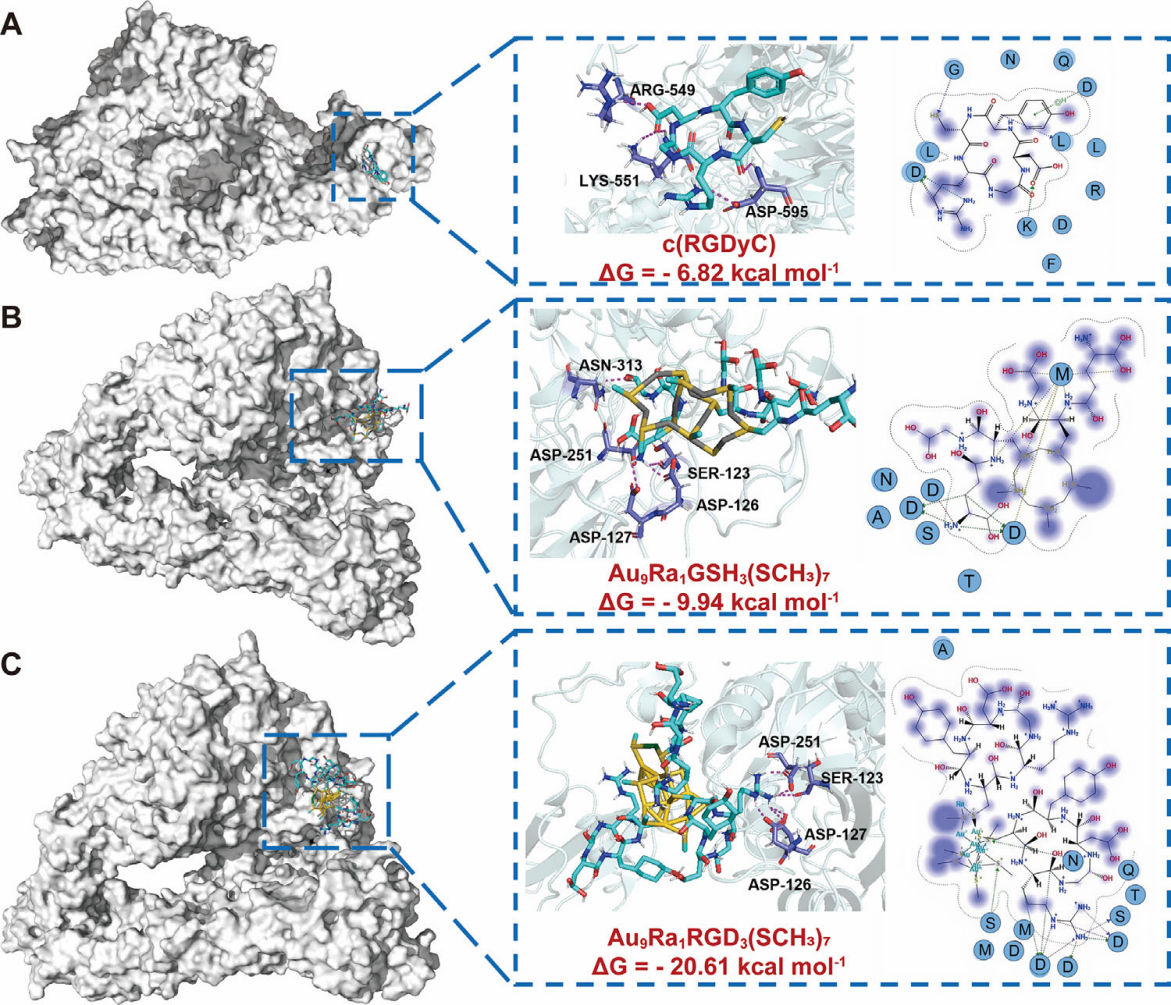
Molecular docking study of the AuNCs. (A–C) Stable binding poses of c(RGDyC) (A), Ra@AuNCs (B), and Ra@AuNCs‐RGD (C) with the α_v_β_3_ integrin receptor. The binding free energy for c(RGDyC), Ra@AuNCs, and Ra@AuNCs‐RGD was predicted as −6.82, −9.94, and −20.61 kcal mol^−1^, respectively. 2D interaction diagrams provide information about the AuNCs’ exposure toward binding with α_v_β_3_ integrin receptor. The active site residues are represented as follows: polar residues in light purple, hydrophobic residues in green, acidic residues with a red contour ring, and basic residues with a blue contour ring. Green and blue arrows indicate hydrogen bonding to side chain and backbone atoms, respectively. The cation–π stacking interactions are represented in green dotted lines. The metal ions interaction network is represented in purple dotted lines. The dotted lines are the expression of the area involved in hydrophobic interactions. Light‐purple halos around residues indicate the degree of interaction with the AuNCs (larger and darker halos mean more interaction).

The dominant docking pose revealed that Ra@AuNCs‐RGD exhibited frequent electrostatic interactions with α_v_β_3_, mediated by charged units (highlighted in purple filled circles, Figure 4A–C). Additionally, Ra@AuNCs‐RGD formed hydrogen bonds with specific amino acid residues on the receptor, including ASP‐251, ASP‐127, ASP‐126, ASN‐313, and SER‐334, resulting in a minimum binding free energy of −20.61 kcal mol^−1^. In comparison, plain gold nanocluster Ra@AuNCs and the standalone RGD peptide exhibited significantly weaker binding free energies of −9.94 and −6.82 kcal mol^−1^, respectively. Ra@AuNCs primarily formed hydrogen bonds with ASP‐251 and ASP‐126, while the standalone RGD peptide interacted with ALA‐641, LYS‐532, THR‐556, and SER‐587, as well as with ASP‐595, LYS‐551, and ASP‐457 through hydrogen bonds. The electrostatic interactions of Ra@AuNCs and the standalone RGD peptide with α_v_β_3_ were markedly weaker than those of Ra@AuNCs‐RGD. Therefore, we conclude that the Au_9_RaRGD_3_(SCH_3_)_7_ nanocluster can serve as an effective carrier for ^223^Ra, demonstrating superior targeting specificity toward the α_v_β_3_ integrin receptor compared to small molecules. Critically, targeting molecule modification not only preserves but also actually enhances this specificity.

To validate the computational findings, we performed an in vitro cellular uptake assay to evaluate the α_v_β_3_‐specific targeting capability of RGD‐modified gold nanoclusters. Cell lines with varying integrin receptor expression levels [46, 47, 48] (4T1, high; B16F10, medium; MC38, low) were incubated with ^68^Ga/^223^Ra@AuNCs‐RGD after complete decay of ^223^Ra and ^68^Ga, and intracellular gold content was quantified using inductively coupled plasma mass spectrometry (ICP‐MS) after incubation (Figure S17). The ICP‐MS results revealed cell uptake of ^68^Ga/^223^Ra@AuNCs‐RGD was in line with α_v_β_3_ integrin expression levels; the higher the expression of the receptor, the higher the uptake of the probe (4T1: 49.2 ± 3.7 fg cell^−1^; B16F10: 36.7 ± 3.0 fg cell^−1^; MC38: 17.5 ± 1.5 fg cell^−1^). Pre‐incubation of the cells with free RGD peptide to saturate α_v_β_3_ receptors effectively blocked the cellular uptake of ^68^Ga/^223^Ra@AuNCs‐RGD by 4T1 and B16F10 cells, and no significant difference in uptake was observed in MC38 cells between different treatment groups. To verify the ICP‐MS assay, ^223^Ra@AuNCs‐RGD was labeled with ^68^Ga after complete decay of ^223^Ra, incubated with the three cell lines aforementioned, and the cell uptake of the probes was determined by a gamma counter (Figure S18). The intracellular radioactivity was consistent with intracellular Au contents obtained from ICP‐MS measurements, which further demonstrated the specificity of the probe for the α_v_β_3_ receptor and corroborated the findings from the molecular docking analysis.

To determine the intracellular localization of the AuNCs, the ^68^Ga/^223^Ra@AuNCs‐RGD was fluorescently tagged with FITC after the radionuclides had fully decayed. After incubation with cells, images of confocal microscopy imaging obtained from Z‐stack revealed that the AuNCs were internalized by all cell lines examined (Figure S19). In agreement with the results from ICP‐MS measurements and γ‐counting, the fluorescence intensities of the FITC‐tagged ^68^Ga/^223^Ra@AuNCs‐RGD were markedly higher in 4T1 and B16F10 cells compared to MC38 cells. The nanoclusters were mainly localized in cytoplasmic vesicles and exhibited a heterogeneous distribution within the perinuclear region. This pattern of intracellular distribution is consistent with the known behavior of various nanomaterials [49].

### 2.4 Biocompatibility of the Gold Nanoclusters

Cytotoxicity of gold nanoclusters was first evaluated by cell counting kit‐8 (CCK‐8) assay. The cells (B16F10 melanoma cell line, HK‐2 human renal tubular epithelial cell line, and THP‐1 human monocyte‐derived macrophage) were incubated with ^68^Ga/^223^Ra@AuNCs‐RGD after complete decay of ^223^Ra and ^68^Ga in different concentrations for 24 h. Cell viability gradually decreased with the increase of gold concentrations for all these three cell lines. When the concentration of the nanocluster reached 25 µg mL^−1^, cell viability remained above 90% for all tested lines (B16F10 cells, HK‐2 cells, and THP‐1 cells), indicating the potential of the nanocluster as a carrier of radionuclides for tumor‐targeted therapy (Figure S20).

Subsequently, the acute toxicity of the nanocluster was evaluated. The mice were intravenously injected with ascending doses of ^68^Ga/^223^Ra@AuNCs‐RGD (0, 25, 50, and 75 mg Au kg^−1^ b.w.) after ^68^Ga/^223^Ra had fully decayed. When the doses were below 50 mg Au kg^−1^ b.w., no behavioral abnormalities were observed. However, when the dose increased to 75 mg Au kg^−1^ b.w., the mice exhibited signs of acute toxicity, including passive behavior and tremors. Body weight monitoring over time revealed no significant weight loss at doses up to 50 mg Au kg^−1^ b.w., while mice treated with 75 mg Au kg^−1^ b.w. experienced weight loss within 7 days (Figure S21), indicating that 50 mg kg^−1^ is a safe threshold. To assess potential organ toxicity, major organs (heart, lungs, spleen, liver, and kidneys) were harvested from mice injected with 50 mg Au kg^−1^ b.w. of gold nanoclusters at 1 day, 7 days, and 21 days post‐injection. Histopathological analysis via H&E staining showed no signs of abnormal inflammatory cell infiltration or tissue structure alterations (Figure S22). No significant reduction in organ coefficients was observed, indicating no detectable organ damage (Figure S23).

Finally, comprehensive blood count and serum biochemistry assays were performed to evaluate peripheral blood cells and liver and renal function (Figures S24 and S25). Hepatic function was assessed by measuring alanine aminotransferase (ALT) and aspartate aminotransferase (AST) levels, while kidney function was evaluated using serum urea nitrogen (BUN) and creatinine (CREA) levels. The results revealed that white blood cells (WBCs), red blood cells (RBCs), platelets (PLTs), lymphocyte percentage (W‐SCR), neutrophil percentage (W‐MCR), and monocyte percentage (MONO%), as well as ALT, AST, BUN, and CREA levels, remained within normal ranges at all time points. These findings demonstrate that gold nanoclusters exhibit excellent biological safety when administered at doses ≤50 mg kg^−1^, with no evidence of inflammation or damage to the liver, kidneys, or other major organs, highlighting their potential as safe and effective carriers of radioisotopes for tumor theragnostics.

### 2.5 Tumor Targeting of ^68^Ga/^223^Ra@AuNCs‐RGD

**FIGURE 5:**
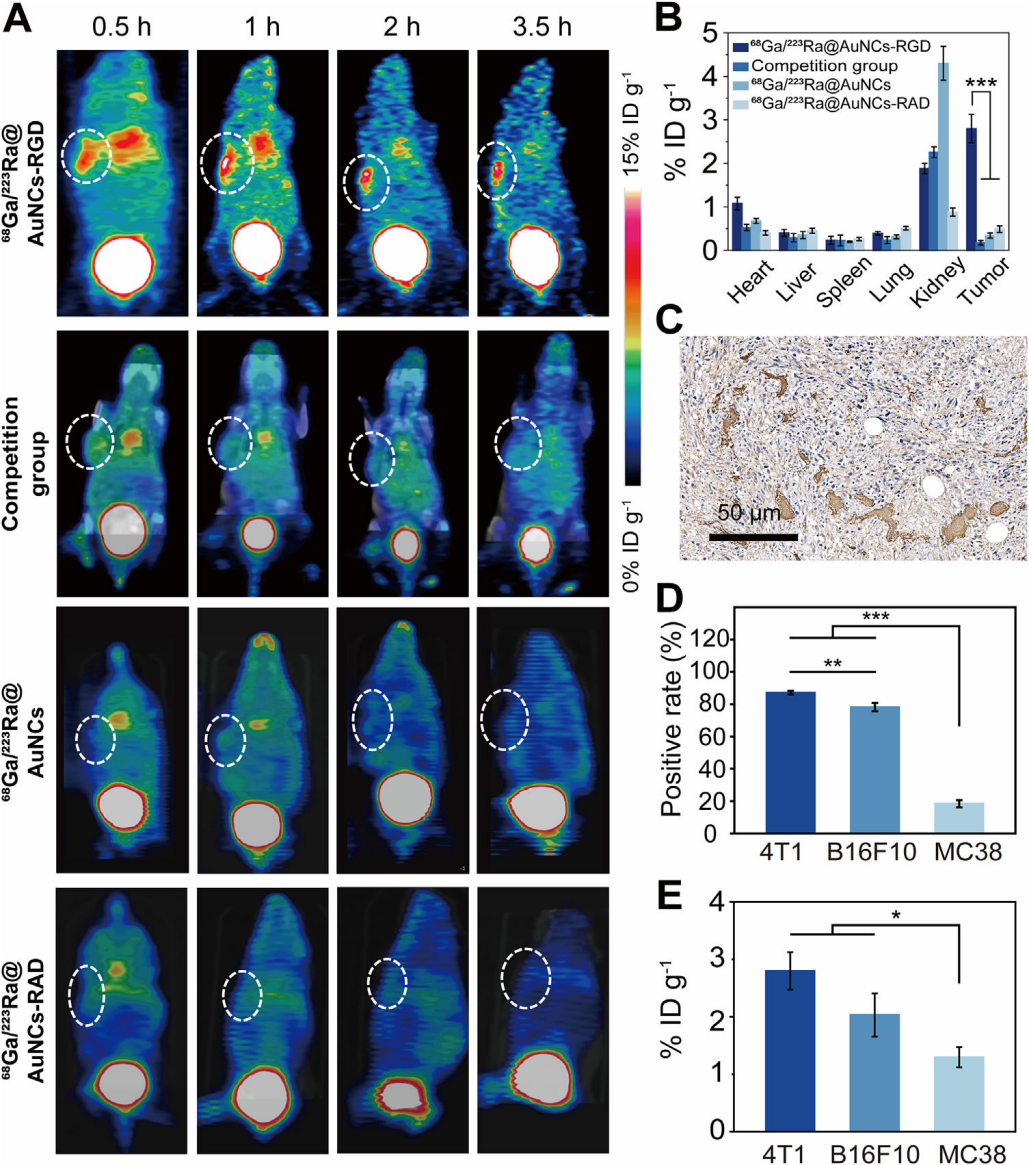
Tumor‐targeting capability of ^68^Ga/^223^Ra@AuNCs‐RGD. (A) PET/CT imaging of the 4T1 tumor‐bearing mice received i.v. injection of ^68^Ga/^223^Ra@AuNCs‐RGD (experimental group), ^68^Ga/^223^Ra@AuNCs‐RGD plus free RGD (competition group), ^68^Ga/^223^Ra@AuNCs (control group), and ^68^Ga/^223^Ra@AuNCs‐RAD (scrambled group) (12.5 mCi kg^−1^ for ^68^Ga, 50 mg Au kg^−1^ b.w.). The white circle denotes the tumor site. (B) Biodistribution of different probes in 4T1 tumor‐bearing mice at 3.5 h p.i. (C) α_v_β_3_ staining of representative tissue from 4T1 tumor. (D) The positive rate of CD61 in different tumor models. (E) Quantitative analysis of tumor uptake of ^68^Ga/^223^Ra@AuNCs‐RGD at 3.5 h p.i. The data represent the mean ± s.d., *n* =  3 biologically independent mice. The error bars represent s.d. values. Analyzed by two‐way ANOVA, followed by Bonferroni's multiple comparisons test.

Next, we investigated whether ^68^Ga/^223^Ra@AuNCs‐RGD could specifically target and efficiently accumulate in tumors and be cleared by the kidneys. B16F10, 4T1, and MC38 subcutaneous tumor models were used for PET/CT imaging. For 4T1 tumor‐bearing mice, the mice were divided into four groups (*n* = 3): ^68^Ga/^223^Ra@AuNCs‐RGD (12.5 mCi kg^−1^ for ^68^Ga, 50 mg Au kg^−1^ b.w.), ^68^Ga/^223^Ra@AuNCs, the competition group (^68^Ga/^223^Ra@AuNCs‐RGD plus free RGD), and ^68^Ga/^223^Ra@AuNCs‐RAD. For B16F10 and MC38 tumor‐bearing mice, the mice were divided into three groups (*n* = 3): ^68^Ga/^223^Ra@AuNCs‐RGD (12.5 mCi kg^−1^ for ^68^Ga, 50 mg Au kg^−1^ b.w.), ^68^Ga/^223^Ra@AuNCs, and the competition group (^68^Ga/^223^Ra@AuNCs‐RGD plus free RGD). PET/CT imaging was performed at different time intervals (0.5, 1, 2, and 3.5 h) post‐injection (Figure 5A and Figures S26A and 27A).

For all these three tumor models, tumor uptake of ^68^Ga/^223^Ra@AuNCs‐RGD increased over time (Figures S29–S31). Three and a half hours after injection, the accumulations by 4T1, B16F10, and MC38 tumors were 2.8 ± 0.3% ID g^−1^, 2.0 ± 0.2% ID g^−1^, and 1.3 ± 0.2% ID g^−1^, respectively. For 4T1 and B16F10 tumors, the PET signal was widely distributed across tumor areas; however, for the MC38 tumor, the signal was mainly distributed at the tumor periphery. However, for all these tumor models, after being blocked with free RGD peptide, tumor uptakes of the probe were significantly reduced (*p* < 0.05 at 3.5 h) (Figure 5B and Figures S26B and S27B). Tumor uptake of the control probe ^68^Ga/^223^Ra@AuNCs and scrambled probe ^68^Ga/^223^Ra@AuNCs‐RAD (for 4T1 tumor) was negligible (Figure 5A and Figure S28). *Ex vivo* biodistribution studies conducted 3.5 h post‐injection were consistent with that in vivo ROI analysis. The maximum tumor‐to‐muscle (T/M) and tumor‐to‐liver (T/L) ratios of ^68^Ga/^223^Ra@AuNCs‐RGD in the 4T1 model were 38.4 ± 4.5 and 7.0 ± 0.8, respectively, significantly higher than those observed in other tumor models (Figure S32). The biodistribution and in vivo metabolism of ^68^Ga/^223^Ra@AuNCs‐RGD were further analyzed (Figures S29–S31). The probe was mainly cleared by kidneys. Liver and spleen sequestrations were marginal, significantly lower than that observed with traditional nanomaterials, which typically exhibit much higher liver and spleen uptake [50, 51].

Finally, immunohistochemical staining of tumor tissues against CD61 was performed for these three tumor models. The 4T1 tumor has the highest α_v_β_3_ integrin expression, followed by the B16F10 tumor. The MC38 tumor has the lowest α_v_β_3_ expression, and the integrin is only expressed in angiogenic vessels (Figure 5C,D and Figures S26C and S27C). The positive rate of CD61 for 4T1, B16F10, and MC38 tumors was 87.01%, 78.51%, and 22.43%, respectively (Figure 5D). These results were consistent with PET imaging of the tumors with ^68^Ga/^223^Ra@AuNCs‐RGD (Figure 5E). Blocking α_v_β_3_ integrin reduced tumor uptake of the RGD probe, and the positive correlation between α_v_β_3_ expression levels and PET signal intensity indicated the specificity of ^68^Ga/^223^Ra@AuNCs‐RGD for α_v_β_3_ integrin [52, 53]. Moreover, ^68^Ga/^223^Ra@AuNCs‐RGD could be cleared by the kidneys but did not accumulate in the liver and spleen, which holds great potential for tumor‐targeted α‐therapy.

### 2.6 In Vivo Targeted α‐Nuclide Therapy

Prior to assessing the in vivo efficacy of targeted α‐nuclide therapy, we first evaluated the anticancer activity of ^68^Ga/^223^Ra@AuNCs‐RGD at the cellular level. 4T1 cells were treated with varying doses of free ^223^Ra, ^68^Ga/^223^Ra@AuNCs, or ^68^Ga/^223^Ra@AuNCs‑RGD for 24 h. Compared with free ^223^Ra and ^68^Ga/^223^Ra@AuNCs, ^68^Ga/^223^Ra@AuNCs‐RGD showed a significantly stronger, radiation‑dose‑dependent inhibitory effect on cancer cells (*p* < 0.001; Figure S33). Even at an ultra‐low radiation dose of ∼50 nCi of ^223^Ra, ^68^Ga/^223^Ra@AuNCs‐RGD induced approximately 53.8% cell death in 4T1 cells following 24 h of co‐incubation. Moreover, colony formation of 4T1 cells was markedly suppressed after treatment with ^68^Ga/^223^Ra@AuNCs‐RGD (^223^Ra dose: 50 nCi), compared with cells treated with normal saline (PBS), free ^223^Ra, or ^68^Ga/^223^Ra@AuNCs (Figure S34).

**FIGURE 6:**
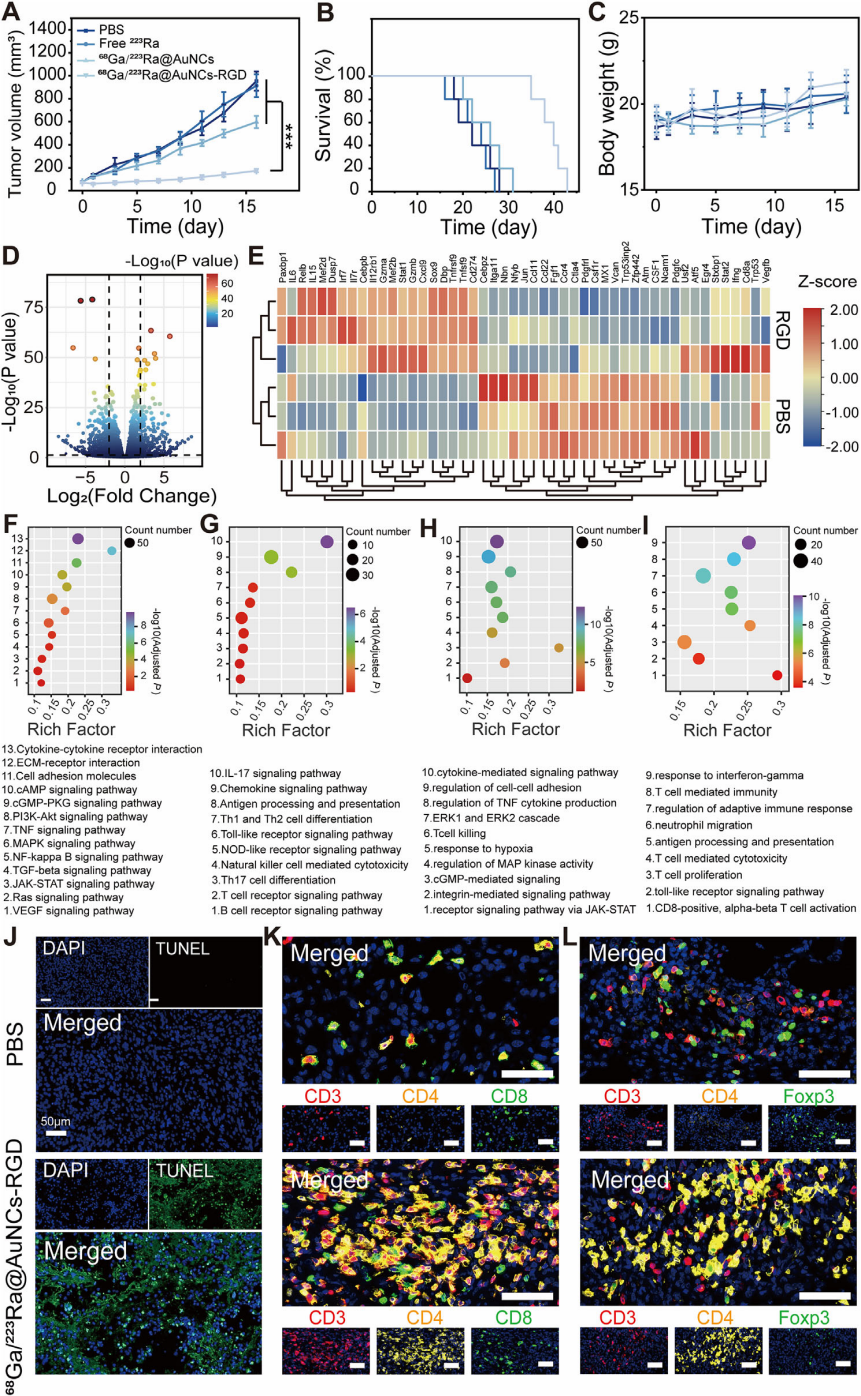
In vivo targeted α‐therapy efficacy and transcriptomic analysis. (A) Relative tumor volume changes in different treatment groups. (B, C). Survival curves (B) and body weight (C) changes of mice after different treatments (mean ± s.d., *n* = 5). (D) Volcano plots of differentially expressed genes between the control group and the ^68^Ga/^223^Ra@AuNCs‐RGD group. (E) Heat maps displaying the differentially transcribed genes of interest between the control group and the ^68^Ga/^223^Ra@AuNCs‐RGD group. (F, G) KEGG enrichment analysis of the differentially expressed TME‐related genes (F) and immune‐related genes (G). (H, I) GO enrichment analysis of the differentially expressed TME‐related genes (H) and immune‐related genes (I). (J) DAPI staining and TUNEL staining of the dissected tumor tissues 2 days after the treatments. Scale bar: 50 µm. (K, L) Multiplex immunohistochemical staining of tumor tissues from 4T1 tumor‐bearing mice treated with PBS and ^68^Ga/^223^Ra@AuNCs‐RGD, with emphasis on the markers CD3 (red), CD4 (yellow), and CD8 (green) (K), or markers CD3 (red), CD4 (yellow), and Foxp3 (green) (L). The nucleus was counterstained with DAPI. Scale bar = 20 µm. Data are presented as mean ± s.d.

Compared to β‐emitters such as ^177^Lu and ^89^Sr, α‐emitters have a much higher LET (80–100 keV µm^−1^) and exhibit significantly higher cell‐killing efficiency by inducing DNA double‐strand breaks. However, the absence of a stable labeling chelating agent has limited the delivery of ^223^Ra to non‐bone tumors. Building on the previous findings that ^68^Ga/^223^Ra@AuNCs‐RGD had high tumor accumulation and a favorable pharmacokinetics profile, we next assessed its potential for targeted ^223^Ra therapy. 4T1 tumor‐bearing mice were randomly divided into control (PBS injection), free ^223^Ra (^223^RaCl_2_ injection), ^68^Ga/^223^Ra@AuNCs, and ^68^Ga/^223^Ra@AuNCs‐RGD groups with ten mice in each group. The probes were intravenously injected with 0.25 mCi kg^−1^ b. w. of ^223^Ra. Due to its non‐specific tumor targeting, the treatment effect of free ^223^Ra was not markedly different from that of the control group (PBS injection) (Figure 6A). ^68^Ga/^223^Ra@AuNCs‑RGD significantly suppressed tumor growth, whereas ^68^Ga/^223^Ra@AuNCs only marginally inhibited tumor progression (*p* < 0.001; Figure 6A). Meanwhile, the median survival times of mice after treatment with PBS, free ^2^ ^2^ ^3^Ra, ^68^Ga/^223^Ra@AuNCs, and ^68^Ga/^223^Ra@AuNCs‑RGD were 24, 22, 26, and 40 days, respectively (Figure 6B).

4T1 tumor has a highly immunosuppressive tumor microenvironment (TME), mimicking key features of aggressive human triple‐negative breast cancer (TNBC) [54]. This immunosuppression is a major reason for its rapid growth, metastasis, and resistance to many immunotherapies [55]. To further explore the impact of targeted α‐therapy with ^68^Ga/^223^Ra@AuNCs‐RGD on the immunosuppressive TME, tumor tissues were collected for transcriptomic analysis two days after injection. Principal component analysis revealed distinct scatter plots comprising two groups: the PBS group and the ^68^Ga/^223^Ra@AuNCs‐RGD group (Figure S35). Within each group, three samples were closely clustered, indicating high repeatability. Furthermore, there was significant variation observed between the two groups. A total of 23,020 genes were analyzed, identifying 2064 differentially expressed genes (DEGs) (absolute fold change ≥2, *p* values < 0.05), including 697 upregulated and 1367 downregulated genes (Figure 6D). This suggests that ^68^Ga/^223^Ra@AuNCs‐RGD significantly modulates the TME following alpha therapy. Our primary focus was on the expression patterns that might influence cell death, metastasis, and interactions with the immune system, as these are critical indicators of the therapeutic response. Consequently, we conducted a more detailed analysis of certain differential genes, as illustrated in the heat map (Figure 6E). Notably, relative to the PBS group, the Atm and Nbn genes were significantly downregulated, which impaired the repair of damage following DNA double‐strand breaks induced by α‐particles and effectively promoted tumor cell death [56, 57]. Concurrently, the downregulation of PDGFR AND PDGFC significantly inhibited tumor metastasis, which is critical for favorable therapeutic outcomes [58]. Additionally, the treatment group exhibited upregulated genes such as Cxcl9, IFNg, Cd8a, Il12rb1, Il7r, IL‐6, Gzmb, and Gzma, alongside downregulated genes including Vcan, Ctla4, Itga11, and Csf1r. This expression profile indicates enhanced cytotoxic T lymphocyte (CTL) activity and reduced regulatory T cell (Treg) presence, fostering an immune microenvironment conducive to tumor clearance [59, 60].

To further explore the effect of ^223^Ra α‐therapy on tumor cells, we conducted Kyoto Encyclopedia of Genes and Genomes (KEGG) and Gene Ontology (GO) pathway enrichment analyses on the DEGs (Figure 6F–I). ^68^Ga/^223^Ra@AuNCs‐RGD group showed significant enrichment in cellular interactions and associated signaling pathways, including cytokine‐cytokine receptor interaction, PI3K‐Akt, MAPK, cAMP, cAMP‐PKG, TNF, and NF‐kappa B signaling pathways. Among these, the cytokine‐cytokine receptor interaction pathway was the most enriched, accounting for 8.07% of the total. Notably, we observed a marked upregulation of Tnfrsf9/Tnfsf9 receptor ligands and a significant downregulation of ccl22/ccr4 receptor ligand chemokines. These findings suggest that the treatment effectively activates antigen‐presenting cells (APCs), thereby enhancing anti‐tumor immunity [61, 62]. We also noticed the enrichment of immune pathways, including chemokine signaling, IL‐17 signaling, antigen processing and presentation, Th1 and Th2 cell differentiation, and the Toll‐like receptor signaling pathway, among others. The study revealed that treatment with ^68^Ga/^223^Ra@AuNCs‐RGD, in comparison to the control group, led to an upregulation of genes associated with the “positive regulation of innate immune response” and “positive regulation of adaptive immune response.” The GO pathway enrichment analysis revealed the activation of several critical pathways within the TME (Figure 6G–I), including “regulation of cell‐cell adhesion,” “cytokine‐mediated signaling pathway,” “regulation of TNF cytokine production,” “receptor signaling pathway via JAK‐STAT,” “regulation of MAP kinase activity,” and “cGMP‐mediated signaling.” These activated pathways were instrumental in modulating the TME, aligning with the KEGG analysis results. Additionally, H&E and terminal deoxynucleotidyl transferase dUTP nick end labeling (TUNEL) staining confirmed that mice treated with ^68^Ga/^223^Ra@AuNCs‐RGD exhibited significant tumor cell damage and extensive apoptosis (Figure 6J).

To corroborate the immune activation observed in the transcriptomic data, tumors were collected and subjected to analysis via flow cytometry (FCM) and immunohistochemical staining. FCM results demonstrated that compared to the control (PBS group), ^68^Ga/^223^Ra@AuNCs‐RGD significantly increased intratumoral CD4^+^ T cell (7.85% vs. 2.89%) and CD8^+^ T cell infiltration (12.63% vs. 9.01%) and reduced immunosuppressive Tregs populations (4.47% vs. 11.43%) (Figures S36 and S37). The findings from immunohistochemical analyses corroborated the flow cytometry results (Figure 6K,L), indicating an activated antitumor immune microenvironment. Additionally, serum cytokine levels, including TNF‐α, IFN‐γ, IL‐10, and TGF‐β, were also evaluated. After treatment with ^68^Ga/^223^Ra@AuNCs‐RGD, there was a significant elevation in TNF‐α and IFN‐γ levels, while IL‐10 and TGF‐β levels were notably decreased (Figure S38). The transcriptomic data and flow cytometry analysis indicate that ^68^Ga/^223^Ra@AuNCs‐RGD modulates critical immune pathways, such as cytokine‐cytokine receptor interactions and Th1/Th2 differentiation, thereby enhancing immune activity and reducing Treg infiltration. These alterations prime the TME for synergistic interactions with cancer immunotherapy, including immune checkpoint inhibitors (ICIs) or anti‐PD‐1/PD‐L1 antibodies. Collectively, these findings indicated that ^68^Ga/^223^Ra@AuNCs‐RGD is very promising as a versatile theranostic agent with potential application for early‐line therapy in aggressive TNBC and other solid tumors exhibiting similar immunosuppressive TME characteristics.

Following successful demonstration of the efficacy of TAT using ^68^Ga/^223^Ra@AuNCs‐RGD, we investigated potential adverse effects of the therapy. Major organs and tumor tissues were collected 16 d after i.v. injection of ^68^Ga/^223^Ra@AuNCs‐RGD. Throughout the treatment period, no significant weight loss was observed in the mice, indicating the safety of the therapy (Figure 6C). ^223^RaCl_2_ (Xofigo) has been approved for palliative treatment of hormone‐refractory prostate cancer with bone metastasis due to the high affinity of free ^223^Ra^2+^ to the bone. As shown in H&E stains, free ^223^Ra could induce severe apoptosis of bone marrow cells, leading to marrow damage. The expression levels of OCN and TRAP were markedly elevated, which may be attributed to free ^223^Ra‐induced damage to osteoblasts and osteoclasts within bone tissue [63, 64, 65]. The damage appears to trigger local inflammatory responses and a compensatory increase in bone metabolism. These effects were not observed in both the PBS group and the ^68^Ga/^223^Ra@AuNCs‐RGD group, suggesting stable doping of ^223^Ra in AuNCs (Figure S39). Histological analysis of the heart, liver, spleen, lung, and kidney revealed no notable tissue damage (Figure S40). Complete blood count parameters in the treatment groups remained within normal ranges (Figure S41). Blood biochemistry tests for liver and kidney functions also showed no significant changes in key parameters, including ALT, AST, BUN, and CREA (Figures S42–S43). All the findings suggest that the TAT with ^68^Ga/^223^Ra@AuNCs‐RGD holds great potential as a safe and effective approach for cancer treatment.

### 2.7 Pharmacokinetics and Tumor Dose Deposition of ^68^Ga/^223^Ra@AuNCs‐RGD

**FIGURE 7:**
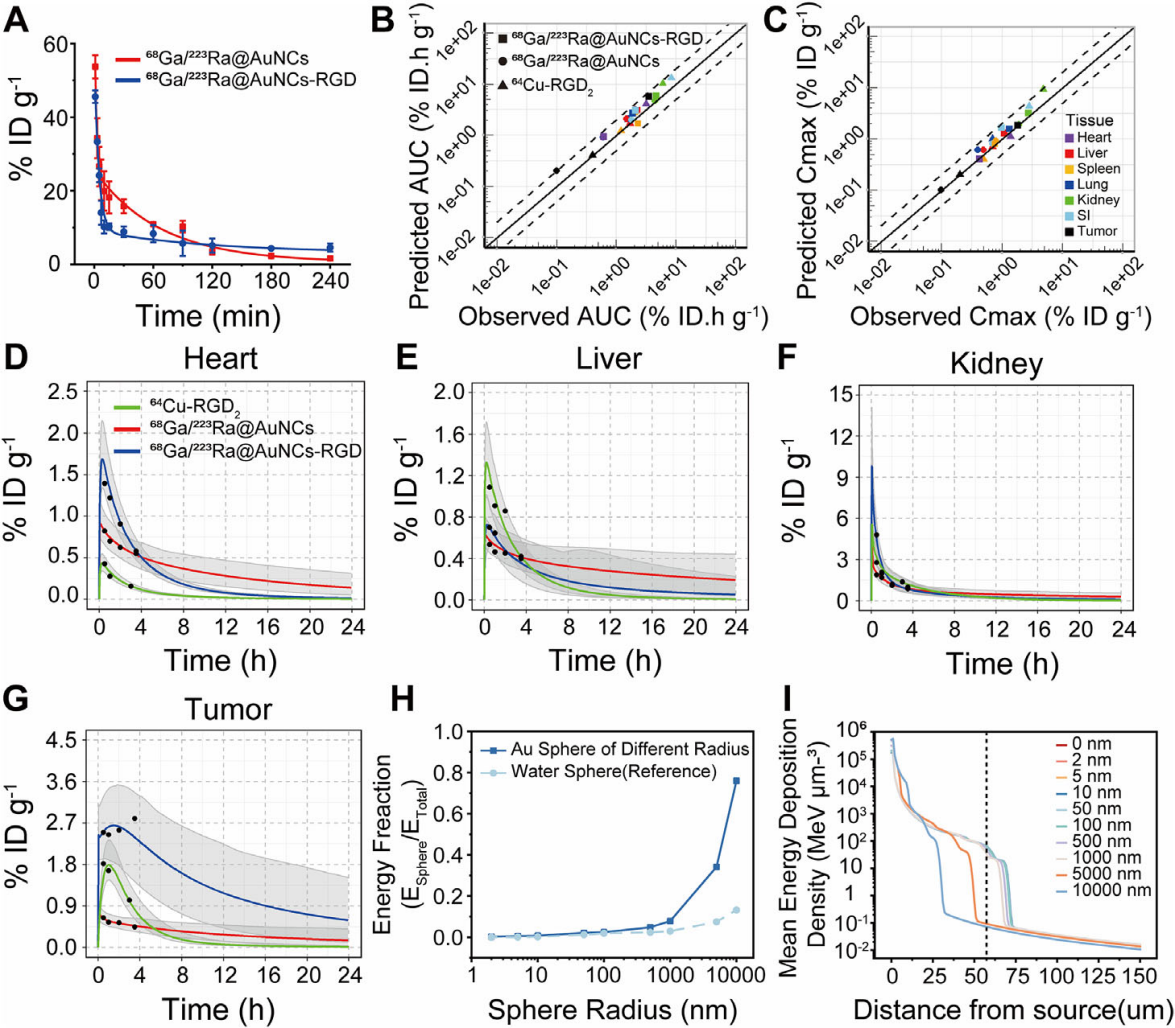
Pharmacokinetics profile and dosimetric analyses of the ^68^Ga/^223^Ra‐labeled AuNCs. (A) The blood half‐life of the ^68^Ga/^223^Ra‐labeled AuNCs determined by a two‐compartment pharmacokinetic model. (B, C) The simulated concentration–time curve (AUC) and the maximum concentration (Cmax) of the ^68^Ga/^223^Ra‐labeled AuNCs. (D–I) The biodistribution of the ^68^Ga/^223^Ra‐labeled AuNCs in heart (D), liver (E), kidney (F), and tumor (G) (*n* =  3). The dots indicate the observed value, the curves indicate predicted means, and the shaded area indicates the 95% prediction interval. (H) Fraction of energy deposited in the gold shells or water shells. (I) Evolution of the energy deposition density with distance from the source.

First, we determined their blood circulation half‐lives in 4T1 tumor‐bearing mice using a two‐compartment pharmacokinetic model [66]. Both ^68^Ga/^223^Ra@AuNCs‐RGD and ^68^Ga/^223^Ra@AuNCs had short distribution half‐lives (*t*_1_/_2_α) of 1.6 and 2.7 min, respectively, indicating rapid tissue distribution of the nanoclusters after i.v. injection (Figure 7A). However, the clearance half‐life (*t*_1_/_2_β) of ^68^Ga/^223^Ra@AuNCs‐RGD was 139.4 min, significantly longer than that of ^68^Ga/^223^Ra@AuNCs (65.2 min), suggesting that RGD modification extends the in vivo circulation time of the nanoclusters, thereby increasing their systemic availability.

PBPK modeling approach is a mechanism‐based computational modeling method that simulates absorption, distribution, metabolism, and excretion (ADME) of substances in an organism. It integrates physiological structures with the physicochemical properties of nano drug delivery systems to quantitatively describe kinetic processes in major organs and tumors [67]. Previously, we have developed a PBPK model to assess the biodistribution and metabolism of RGD peptide‐functionalized gold nanoparticles and nanorods in tumor‐bearing mice [68]. To understand the pharmacokinetic profile of the AuNCs, we further optimized model parameters in this study. This optimized model was then used to characterize AuNCs’ disposition in tumor‐bearing mice and analyze their tissue accumulation patterns. Moreover, the pharmacokinetics of the ^64^Cu‐labeled RGD dimer previously reported was also simulated as a comparison [69]. The model's predictive accuracy was assessed using the mean fold error, defined as the ratio of predicted to observed pharmacokinetic (PK) parameters.

Simulation results showed that predicted PK parameters, including the area under the concentration–time curve (AUC) and maximum concentration (Cmax), aligned well with observed data (within a 0.5 to 2.0 ratio) in most cases, validating the model's reliability (Figure 7B,C). The validated PBPK model was then used to simulate the distribution of the AuNCs in a virtual mouse population (weights: 15–25 g) within 24 h (Figure 7D–G and Figure S44). Experimental observations of probe concentrations in various organs closely matched the simulated values, falling within the 95% prediction interval, confirming the model's accuracy. ^68^Ga/^223^Ra@AuNCs‐RGD exhibited higher concentrations in the heart, liver, spleen, lungs, kidneys, and tumor compared to ^68^Ga/^223^Ra@AuNCs, which is consistent with the biodistribution of the AuNCs determined by ROI from PET imaging. While ^68^Ga/^223^Ra@AuNCs‐RGD initially showed higher concentrations, faster clearance, in the liver and kidneys, and its levels dropped below that of ^68^Ga/^223^Ra@AuNCs after two hours. ^68^Ga/^223^Ra@AuNCs‐RGD demonstrated a 3.3‐fold higher Cmax and a 3.8‐fold greater AUC in tumors within 4 h compared to ^68^Ga/^223^Ra@AuNCs. Furthermore, ^68^Ga/^223^Ra@AuNCs‐RGD showed a 1.5‐fold increase in Cmax and a 2.2‐fold increase in AUC in tumors within the same period when compared to the RGD dimer. These findings underscore the enhanced tumor‐targeting efficacy and extended tumor retention of ^68^Ga/^223^Ra@AuNCs‐RGD relative to peptide–drug conjugates, highlighting its potential as a vector for the efficient α‐therapy. These data suggest that ^68^Ga/^223^Ra@AuNCs‐RGD is stable in the blood and highly effective in the target delivery of ^223^Ra to the tumor tissues, consistent with its enhanced therapeutic efficacy.

In light of the potential for gold nanoparticles (AuNPs) as ^223^Ra carriers to absorb a portion of α‐particle energy, we utilized Monte–Carlo simulations to model the energy deposition of carriers within tumor tissues. Water was selected as the tissue‐equivalent material [70], and a ^223^Ra radioactive source was encapsulated within AuNPs of varying thicknesses (2 nm, 5 nm, 10 nm, 50 nm, 100 nm, 500 nm, 1 µm, 5 µm, and 10 µm; Figure S45). The findings indicated that AuNPs are capable of absorbing some α‐particle energy; however, when the gold size is less than 10 nm, the energy absorbed by the AuNPs constitutes 0.75% of the total energy (0.17% of the total energy absorbed in a water shell with 10 nm thickness; Figure 7H), which would not interfere with the energy deposition in tumors. In addition, high deposition energy density can be maintained within a range of 57 µm when encapsulated in a gold shell with 10 nm thickness (Figure 7I). Considering that the particle size of ^68^Ga/^223^Ra@AuNCs‐RGD is approximately 2 nm, it is insufficient to significantly affect the α‐energy deposition of ^223^Ra at tumor sites.

## 3 Conclusion

Targeted α‐therapy with ^223^Ra is hindered in clinical practice due to the lack of stable chelating agents capable of withstanding its high recoil energy and the five decay daughters generated during decay. To address this, we propose encapsulating ^223^Ra and its decay progeny within gold nanoclusters. We engineered RGD‐functionalized, dual‐labeled Au_10–12_ nanoclusters (^68^Ga/^223^Ra@AuNCs‐RGD) for tumor‐targeted α‐therapy. DFT simulations were performed to thoroughly investigate the stable doping mechanism of ^2^ ^2^ ^3^Ra and its progeny within the AuNCs. The resulting ^68^Ga/^223^Ra@AuNCs‐RGD demonstrated specific targeting of α_v_β_3_ integrin, efficient tumor accumulation, and predominantly renal clearance with minimal retention in the liver and spleen. In therapeutic studies, ^68^Ga/^223^Ra@AuNCs‐RGD eradicated tumors, significantly activated antitumor immune responses, and exhibited no observable adverse effects. PBPK modeling revealed that the nanoclusters exhibited an extended blood half‐life, underwent primary renal clearance, and showed prolonged retention at tumor sites. This study establishes a foundational framework for developing novel ^223^Ra‐labeled α‐radiopharmaceuticals and demonstrates significant promise for clinical translation.

## 4 Methods

### 4.1 Synthesis of AuNCs and RGD Peptide‐Functionalized AuNCs (AuNCs‐RGD)

All chemicals were purchased from Sigma‐Aldrich (Shanghai, China) unless otherwise specified. Both AuNCs and AuNCs‐RGD were synthesized by a one‐step reduction method. In a typical procedure, 99 µL of HAuCl_4_ (0.243 M) was added to 5 mL of an aqueous glutathione solution (GSH, 7.2 mM) under rapid stirring at room temperature for 5 min. The pH of the solution was then adjusted to neutral (pH = 7.4) using NaOH solution (1 M). The mixture was stirred in a water bath at 45°C for 1 h. The resulting AuNCs were collected by ultrafiltration (3 kDa; Millipore) and washed three times with phosphate‐buffered saline (PBS, pH = 7.4). For AuNCs‐RGD, the synthesis procedure was similar to that of AuNCs, except that a mixture of 5 mL of GSH solution (7.2 mM) and 1 mL of c(RGDyC) solution (12 mM; Shanghai Apeptide Co., Ltd, Shanghai, China) was used.

To label ^68^Ga, the AuNCs were further functionalized with DOTA. Specifically, 1 mL of DOTA‐NHS solution (18 mM, in DMSO) was added to 5 mL of an aqueous glutathione solution (GSH, 7.2 mM) and incubated overnight. Then the mixture was used to synthesize the AuNCs following the same procedure described above. The resulting DOTA‐functionalized AuNCs (DOTA@AuNCs) and AuNCs‐RGD (DOTA@AuNCs‐RGD) were obtained after ultrafiltration.

### 4.2 Characterizations

TEM analysis was performed using a field emission transmission electron microscope (∗/JEM‐2100 F; JEOL, Tokyo, Japan) operated at 200 kV. Hydrodynamic diameter was determined through DLS using a Malvern Zetasizer (NANO‐ZS; Malvern Instruments Ltd., Worcestershire, UK). The Au contents were measured using an atomic absorption spectrophotometer (AAS; Z‐2000, Hitachi, Tokyo, Japan). The mass spectra were performed with a Waters Xevo G2‐XS QT of mass spectrometer. Colloidal stability of AuNCs was evaluated by incubating the clusters (1 mg mL^−1^, 1:4, v/v) in mouse serum at 37°C, with particle size changes monitored by DLS at specific time intervals.

### 4.3 Synthesis of ^223^Ra‐Labeled AuNCs (^223^Ra@AuNCs, ^223^Ra@AuNCs‐RGD, and ^223^Ra@AuNCs‐RAD)

^223^Ra (*t*_1/2_ = 11.4 days) was obtained from radium [^223^Ra] chloride injection (Xofigo; Bayer Pharma, Germany), and ^223^Ra‐labeled AuNCs were synthesized through in situ doping of ^223^Ra during synthesis of AuNCs. In a typical procedure, radium [^223^Ra] chloride injection (29 µCi, 1 mL) and HAuCl_4_ (0.243 M, 99 µL) were added to the mixture of GSH and GSH‐DOTA (6 mL) with rapid stirring at room temperature for 5 min. NaOH solution (1 M) was then added to adjust pH to neutral (pH = 7.4). Subsequently, the mixtures were stirred in a water bath at 45°C for 1 h. Then the ^223^Ra‐labeled AuNCs (^223^Ra@AuNCs) were obtained through ultrafiltration described above. The RGD‐functionalized, ^223^Ra‐labeled AuNCs (^223^Ra@AuNCs‐RGD) were prepared following the same procedure as that for ^223^Ra@AuNCs, except that a mixture of GSH and GSH‐DOTA mixture solution (6 mL) and c(RGDyC) solution (12 mM, 1 mL; Shanghai Apeptide Co. Ltd) was used. The RAD‐functionalized, ^223^Ra‐labeled AuNCs (^223^Ra@AuNCs‐RAD) were prepared following the same procedure as that for ^223^Ra@AuNCs, except that a mixture of GSH and GSH‐DOTA mixture solution (6 mL) and c(RADyC) solution (12 mM, 1 mL; Shanghai Apeptide Co. Ltd) was used.

### 4.4 Labeling ^223^Ra@AuNCs With ^68^Ga

^68^Ga (*t*_1/2_ = 67.6 min, 10 mCi in 1 mL of 0.05 M HCl) was eluted from a ^68^Ge/^68^Ga generator (ITG, Berlin, Germany). For labeling, ^68^Ga (10 mCi, 1 mL) was added to either ^223^Ra@AuNCs, ^223^Ra@AuNCs‐RGD, or ^223^Ra@AuNCs‐RAD suspension, and then heated at 100°C for 20 min. The labeled products, ^68^Ga/^223^Ra@AuNCs or ^68^Ga/^223^Ra@AuNCs‐RGD, were washed and collected by ultrafiltration (3 kDa; Pall).

### 4.5 Radio‐Labeling Performance Characterizations

The radioactivity of ^223^Ra was measured using a gamma counter (GC‐1500; USTC Chuangxin Co., Ltd., China). The radioactivity of ^68^Ga was measured using a radioactivity meter (HD‐175A; Heyiqitx, Beijing, China). The radiolabeling efficiency was calculated as the ratio of the radioactivity of the nanoclusters to the total radioactivity initially added.

For estimating the radiolabeling stability, ^223^Ra@AuNCs and ^68^Ga/^223^Ra@AuNCs were incubated with mouse serum at 37°C for various time periods. Following incubation, the radioactive AuNCs were collected by ultrafiltration (7000 rpm, 10 min). The radioactive stability of ^223^Ra was calculated by dividing the radioactivity retained on the AuNCs by the total sample radioactivity. The radiolabeling stability of ^68^Ga in ^68^Ga/^223^Ra@AuNCs was determined using instant radio‐thin layer chromatography (rTLC), with 20 mM citric acid as the developing solvent.

### 4.6 Structural Search and Optimization

AIRSS is a robust and highly parallel computational tool that generates plausible crystal configurations randomly and subsequently optimizes them to reach local energy minima [38], which has been used to predict the most promising structure of Au_10_(SCH_3_)_10_ and Ra@Au_9_(SCH_3_)_10_. The search for each composition was confined to a reduced formula consisting of 60 atoms. For each composition, more than 2000 random structures were generated and subsequently optimized. The plane wave DFT code CASTEP, which employs core‐corrected ultrasoft pseudopotentials as outlined in the built‐in QC5 library, was employed for the search process, with a plane‐wave cutoff energy set at 350 eV, and Brillouin zone integration was performed using a Monkhorst–Pack k‐point grid with a spacing of 0.07 Å^−^^1^ (corresponding to 2π × 0.07 in reciprocal space). Following the structural search results, the investigation was expanded to include a series of the cluster compounds.

The DFT calculations were carried out using the Vienna Ab‐initio Simulation Package (VASP version 6.1.2). The Perdew–Burke–Ernzerhof (PBE) functional within the generalized gradient approximation was used to model the exchange‐correlation energy. The ionic cores were described by projector‐augmented wave pseudopotentials, with an energy cutoff of 450 eV. Grimme's DFT‐D3 method was incorporated to implement the Van der Waals correction. Atomic positions are optimized with the convergence of force less than 0.03 eV Å^−1^ with the electronic relaxation threshold of 10^−5^ eV. Monkhorst–Pack *k*‐points of 1 × 1 × 1 were applied for all the calculations. Then, the original Ra atom was replaced with the atoms of daughter nuclides (Rn, Po, Pb, Bi, and Tl). And the optimized configurations having the lowest energy were used for the subsequent analysis and calculation.

### 4.7 DFT Simulation Calculation

Formation energy (*∆E*) was defined as [71]

$$\Delta E=E_{\text{Ra-defect}}-E_{\mathrm{perfect}}+E_{\mathrm{Au}}-E_{\mathrm{Ra}}$$

where *E*_Ra‐defect_ is the energy of the Ra@Au_9_ cluster, *E*_perfect_ is the energy of the Au_10_ cluster, and *E*_Au_ and *E*_Ra_ are the energies of the Au and Ra atoms, respectively.

Relative energies was defined as [72]

$$\Delta E=E_{M@\mathrm{Au}9}-\left(E_{\mathrm{Au}10}+E_{M}-E_{\mathrm{Au}}\right)$$

where *E*_M@Au9_ is the energy of the M@Au_9_ cluster, *E*_Au10_ is the energy of the Au_10_ cluster, *E_M_* and *E*_Au_ are the energies of a single M atom in the ground‐state structure of the pure M element, and a single Au atom in the ground‐state structure of the pure Au element, respectively.

The binding energy (*E*_b_) was defined as [73]

$$E_{b}=E_{\mathrm{Au}9}+E_{M}-E_{M@\mathrm{Au}9}$$

where *E*_Au9_ is the energy of the Au_9_ frame, *E*_M_ is the energy of the heteroatom (M) atom, and *E*_M@Au9_ is the energy of heteroatom (M)‐doping clusters, respectively.

The bonding/antibonding population between the S and heteroatom doping in Au_10_ structure was analyzed using COHP [74].

Bader charge analysis was used to estimate charge transfer in the simulations [44]. The structure figures of Bader charge transfer were drawn by VESTA.

PDOS of the heteroatom‐doped Au_10_ clusters was calculated using the first‐principles calculations using the VASP.

The DMol^3^ code implemented in BIOVIA Materials studio software was used for the simulation of the HOMO and LUMO energy gap (*E*_gap_) for the heteroatom‐doped Au_10_ clusters [40]. The generalized‐gradient approximation approach of the PBE function was used for the structural optimization and electronic properties calculations, with the convergence tolerance of 1 × 10^−5^ Ha, 0.002 Ha Å^−1^, and 0.005 Å for the energy, force, and displacement, respectively.

The *E*_gap_ was defined as

$$E_{\mathrm{gap}}=E_{\mathrm{LUMO}}-E_{\mathrm{HOMO}}$$

where *E*_LUMO_ and *E*_HOMO_ are the energies of the LUMO and HOMO orbitals, respectively.

### 4.8 Molecular Docking and Analysis of Binding‐Pocket Residues

Molecular docking studies were carried out using the AutoDock 4.2 tool to predict the preferred binding pose and binding sites of AuNCs [75]. The nanocluster models were constructed using Materials Studio, and their geometry was optimized by combining the use of VASP, ORCA 6.0.0 [76], and Multiwfn_dev_3.8 [77]. The crystal structure of the α_v_β_3_ integrin receptor was obtained from the Protein Data Bank with PDB ID 1L5G [78]. Gasteiger charges were automatically added to both protein and ligand, with torsional bond energies set to zero. The final docking results were analyzed using Chimera [79] (www.cgl.ucsf.edu/chimera/) and PyMOL (https://pymol.org/2/), and a protein–ligand interaction diagram was generated.

### 4.9 Cell Culture

All cell lines were obtained from the Cell Bank of Chinese Academy of Sciences (Shanghai, China). Cell line authentication was performed by short tandem repeat profiling, and routine mycoplasma testing confirmed the absence of contamination. The 4T1 (RRID: CVCL_0125), MC38 (RRID: CVCL_B288), B16F10 (RRID: CVCL_0159), and HK‐2 cell (RRID: CVCL_0302) lines were cultured in DMEM supplemented with 10% FBS and 1% penicillin–streptomycin under humidified conditions at 37°C with 5% CO_2_. The THP‐1 cell line (RRID: CVCL_0006) was cultured in RPMI 1640 with 10% FBS and 1% penicillin–streptomycin under the same conditions.

### 4.10 Cell Uptake of RGD‐Modified AuNCs

To assess cell uptake, B16F10, 4T1, and MC38 were seeded in 12‐well plates (2 × 10^5^ cells per well) and incubated with ^68^Ga/^223^Ra@AuNCs, ^68^Ga/^223^Ra@AuNCs‐RGD, or ^68^Ga/^223^Ra@AuNCs‐RGD (200 µg Au mL^−1^) plus free c(RGDyC) peptide (1.68 mM, 25 µL) for 1 h. After the radioisotopes were fully decayed, cellular uptake was quantified by ICP‐MS measurement of Au content. AuNCs (post complete decay of ^223^Ra). Moreover, after complete decay of ^223^Ra, ^223^Ra@AuNCs was labeled with ^68^Ga, and then B16F10, 4T1, and MC38 were incubated with ^68^Ga/^223^Ra@AuNCs, ^68^Ga/^223^Ra@AuNCs‐RGD, or ^68^Ga/^223^Ra@AuNCs‐RGD (200 µg Au mL^−1^) plus free c(RGDyC) peptide (1.68 mM, 25 µL) for 1 h. The intracellular radioactivity of ^68^Ga was countered by a gamma counter.

### 4.11 Confocal Fluorescence Cell Imaging

To investigate the cellular uptake and intracellular localization of the AuNCs, RGD‐modified AuNCs were visualized using confocal laser scanning microscopy. Fluorescent tagging was achieved by conjugating FITC‐NHS ester (Shanghai Apeptide Co. Ltd) to ^68^Ga/^223^Ra@AuNCs or ^68^Ga/^223^Ra@AuNCs‐RGD (following the complete decay of radionuclides, Au:FITC‐NHS ester  =  4:1 as a molar ratio) for 1 h at room temperature. The FITC‐tagged nanoclusters were subsequently purified via ultrafiltration.

B16F10, 4T1, and MC38 cells were seeded into chamber slides at a density of 5 × 10^4^ cells per well and cultured for 24 h. The medium was then replaced with FITC‐tagged nanoclusters at an Au concentration of 200 µg mL^−1^ and incubated at 37°C. After 1 h, cells were washed three times with PBS, fixed with 4% paraformaldehyde, and counterstained with DAPI. Fluorescence images were acquired using a Nikon laser scanning confocal microscope.

### 4.12 Cytotoxicity of the Nanoclusters

The cytotoxicity of the nanoclusters was assessed using a CCK‐8 (Beyotime, China) assay, following the manufacturer's instructions. Briefly, B16F10 cells, HK‐2, and THP‐1 cells were seeded in 96‐well plates (1 × 10^4^ cells per well) and exposed to varying concentrations (0–25 µg mL^−1^) of ^68^Ga/^223^Ra@AuNCs or ^68^Ga/^223^Ra@AuNCs‐RGD for 24 h after the radioisotopes were completely decayed.

After incubation, cells were rinsed twice with PBS, followed by the addition of 100 µL of CCK‐8 working solution and an additional incubation for 1 h. Cell viability was quantified by measuring absorbance at 450 nm using a plate reader (PerkinElmer, Baltimore, MD). Viability was expressed as a percentage of the absorbance of cells treated with the AuNCs relative to cells maintained in normal culture medium.

### 4.13 Animal Model

Balb/c mice and C57B/L mice were purchased from SPF Biotechnology Co., Ltd. (Shanghai, China) and were treated in accordance with the protocols approved by the Animal Protection and Care Committee of Shanghai Jiao Tong University. To build the subcutaneous tumor model, each Balb/c mouse (6–8 weeks old) was subcutaneously inoculated with 2 × 10^6^ 4T1 tumor cells into the right armpit, while C57B/L mice (6–8 weeks old) were inoculated with 2 × 10^6^ B16F10 or MC38 tumor cells. Tumor‐bearing mice were considered ready for experiments when tumor size reached 75 mm^3^.

### 4.14 Evaluation of the Acute Toxicity of the Nanocluster

The acute toxicity of the nanocluster was evaluated using normal SPF Balb/c female mice weighing 20–25 g. The mice were injected with different concentrations of ^68^Ga/^223^Ra@AuNCs‐RGD (0, 25, 50, 75 mg Au kg^−1^) after the nuclides complete decay via tail vein. All animals were clinically monitored for a minimum of five weeks for signs of toxicity, which included assessments of body weight, food and water intake, hematology, and hematoxylin and eosin (H&E) staining of main organs.

### 4.15 PET/CT Imaging and Biodistribution

To evaluate the specificity of ^68^Ga/^223^Ra@AuNCs‐RGD for tumors, B16F10, 4T1, and MC38 tumor models were prepared. For 4T1 tumor‐bearing mice, the mice were divided into four groups (*n* = 3): ^68^Ga/^223^Ra@AuNCs‐RGD (12.5 mCi kg^−1^ for ^68^Ga, 50 mg Au kg^−1^ b.w.), ^68^Ga/^223^Ra@AuNCs, the competition group (^68^Ga/^223^Ra@AuNCs‐RGD plus free RGD), and ^68^Ga/^223^Ra@AuNCs‐RAD. For B16F10 and MC38 tumor‐bearing mice, the mice were divided into three groups (*n* = 3): ^68^Ga/^223^Ra@AuNCs‐RGD (12.5 mCi kg^−1^ for ^68^Ga, 50 mg Au kg^−1^ b.w.), ^68^Ga/^223^Ra@AuNCs, and the competition group (^68^Ga/^223^Ra@AuNCs‐RGD plus free RGD). Tumor‐bearing mice were injected via the tail vein. For the competition study, free c(RGDyC) (1.68 mM, 200 µL) was administered 1 h before the injection of ^68^Ga/^223^Ra@AuNCs‐RGD. PET/CT imaging was performed at different time points post injection (0.5, 1, 2, and 3.5 h), and the in vivo biodistribution in multiple tumor models was quantitatively analyzed using ROI functions. Following the conclusion of the experiment, the mice were euthanized, and tumor tissues and major organs (including the heart, liver, spleen, lung, and kidney) were collected, weighed, and counted using a γ counter (GC‐1500; USTC Chuangxin Co., Ltd., China). The bio‐distribution of ^68^Ga/^223^Ra@AuNCs (%ID g^−1^) was then determined. Tumor tissues were stained for integrin α_v_β_3_ (against CD61) to evaluate its expression levels.

### 4.16 In Vitro α‐Therapy Study

To measure the cytotoxicity of ^223^Ra, the CCK‐8 assay was conducted (CCK‐8; Beyotime, China). 4T1 cells were seeded on 96‐well plates (1 × 10^4^ cells per well) and incubated for 24 h. Cells were then treated with free ^223^Ra, ^68^Ga/^223^Ra@AuNCs, and ^68^Ga/^223^Ra@AuNCs‐RGD at different doses of ^223^Ra for 24 h. Cell viability was then evaluated according to the procedure outlined in Section 4.12.

### 4.17 Cell Colony Formation Assay

4T1 cells (1000 cells well^−1^) were inoculated into six‐well plates for 24 h. Subsequently, the cells were treated with the PBS, free ^223^Ra, ^68^Ga/^223^Ra@AuNCs, and ^68^Ga/^223^Ra@AuNCs‐RGD at a dosage of 50 nCi for ^223^Ra. The culture medium was refreshed every three days. After a 2‐week incubation period, colonies were fixed using glutaraldehyde (6.0% v/v), stained with crystal violet (0.5% w/v), and photographed.

### 4.18 Tumor‐Targeted α‐Therapy

4T1 tumor‐bearing mice were ready for experiments when the tumor size reached about 75 mm^3^. The mice were randomly divided into three groups (*n* = 10), and each group received an intravenous injection of PBS, ^68^Ga/^223^Ra@AuNCs, or ^68^Ga/^223^Ra@AuNCs‐RGD. Due to the high affinity of free ^223^Ra^2+^ to the bone, to demonstrate the stable doping of ^223^Ra in AuNCs, 4T1 tumor‐bearing mice were also treated with ^223^RaCl_2_ injection (Xofigo) for comparison. For each group, the injected doses of ^223^Ra and ^68^Ga were 0.25 and 12.5 mCi kg^−1^, respectively, while the dose of Au was 50 mg kg^−1^ b.w. Two days after injection, one mouse was randomly selected from each group, and tumor tissues were harvested and stained with DAPI and TUNEL.

RNA‐seq of tumor tissues from different treatment groups was performed on day 2 after treatment. RNA extraction, quality control, library construction, and sequencing were performed according to procedures provided by Wuhan MetWare Biotechnology Co., Ltd. DEGs were calculated with the DESeq2 R package. The *p* < 0.05 and fold change |log_2_ fold change|≥ 1 were set as the cutoff criteria. Volcano plots were graphed using the ggplot package in RStudio. A heatmap was generated using Tbtools software [80] (https://github.com/CJ‐Chen/TBtools). KEGG enrichment analysis was performed using the Metware Cloud (https://cloud.metware.cn).

The tumor sizes and body weights of the remaining mice were monitored for 16 days. For the safety evaluation, mice (*n* = 3) were sacrificed, and hematology and H&E staining of main organs were assessed after being treated for 16 days. DAPI/TUNEL and H&E staining and OCN‐ and TRAP‐immunohistochemical staining of the leg bone tissues of mice were performed after being treated for 16 d. Injections of free ^223^Ra alone served as the control. To assess the survivals, mice were treated exactly the same as those for tumor TAT, except that there were five mice in each treatment group. The survival rates were recorded over time. Survival rates were recorded over time and analyzed using Kaplan‐Meier survival analysis.

### 4.19 In Vivo Anti‐Tumor Immune Response

To examine tumor immune cell infiltration, tumors were collected from the PBS and ^68^Ga/^223^Ra@AuNCs‐RGD groups after 7 days of treatment. Tumors were minced and digested with DNase I (0.1 mg mL^−1^) and Liberase TL (0.1 mg mL^−1^) for 1 h to prepare single‐cell suspensions. Subsequently, 1×10^6^ cells were added to each flow cytometry tube for staining with corresponding antibodies against T cells (CD45^+^CD3^+^CD4^+^CD8^+^) and regulatory T cells (Tregs, CD45^+^CD3^+^CD4^+^Foxp3^+^). The antibody included CD45‐BV421 (BioLegend, 103133), CD3‐FITC (BioLegend, 100204), CD4‐APC (BioLegend, 100204), CD8‐PE (BioLegend, 100708), and FOXP3‐PE (BioLegend, 126404). The cell‐antibody mixtures were incubated at 4°C for 1 h in the dark to minimize non‐specific binding and fluorochrome quenching. All stained cells were analyzed using a BD FACSCanto II flow cytometer, and the experimental results were processed with FlowJo software.

To further prove the activating antitumor immunity following ^68^Ga/^223^Ra@AuNCs‐RGD treatments, tumors were processed and analyzed by immunohistochemistry. The expression of microenvironment‐related markers in tumor tissues was detected, including CD3, CD4, CD8, and FOXP3. Additionally, ELISA was performed using the manufacturer's recommendations to detect TNF‐α, IL‐10, TGF‐β, and IFN‐γ.

### 4.20 Half‐Life Assay of the Nanocluster

To evaluate their pharmacokinetics, 50 µL of ^68^Ga/^223^Ra@AuNCs or ^68^Ga/^223^Ra@AuNCs‐RGD (12.5 mCi kg^−1^ for ^68^Ga, 50 mg Au kg^−1^ b.w.) was administered via tail vein injection into 4T1 tumor‐bearing mice. Blood samples were collected from the tail veins at various time points (1, 3, 5, 7, 10, 15, 30, 60, 90, 120, 180, and 240 min), and subsequent procedures were performed in accordance with the established protocols. The half‐lives of the different ^68^Ga/^223^Ra@AuNCs formulations were determined by fitting the data to a two‐compartment i.v. injection model [66].

### 4.21 PBPK Model

PBPK modeling was used to simulate the distribution, metabolism, and elimination characteristics of the ^68^Ga/^223^Ra@AuNCs‐RGD according to our previous report and further optimized [68, 81]. The mice were assumed to be divided into eight tissue compartments: heart, liver, spleen, lung, kidney, tumor, blood, and the remaining organs. The remaining organs of the mice were considered as a single compartment. The heart, liver, spleen, lung, kidney, tumor, and remaining parts were further subdivided into capillary (vascular) and tissue components. A PBPK model was established using R programming, and the distribution of the probe in various tissues and organs at different time points was analyzed. ^64^Cu‐labeled RGD dimer was used as a control based on the literature [69].

### 4.22 Monte–Carlo Simulations

The Geant4 toolkit was utilized to simulate particle transport and investigate the correlation between the thickness of gold nanocarriers and energy deposition in radionuclide therapy [70]. Initially, a macroscopic simulation was performed within a 200 × 200 × 200 µm^3^ water phantom to represent biological tissues, with a spheroidal radioactive source of ^223^Ra, having a radius of 1 nm, positioned at the center [82]. Subsequently, gold shells of varying thicknesses (2 nm, 5 nm, 10 nm, 50 nm, 100 nm, 500 nm, 1 µm, 5 µm, and 10 µm) were constructed surrounding the ^223^Ra radioactive source. The same thickness of water shells was used as a control. For each simulation, the number of radionuclide decays was set to 1 × 10^5^. The complete decay chain to stability for ^223^Ra was generated and transported by the Geant4 physics models according to previous reports [83]. The dose deposition within the gold shells and the water phantom was recorded. Additionally, the variations in the mean energy deposition density of the ^223^Ra radioactive source as a function of the gold shell thickness were calculated.

### 4.23 Statistics

The data are presented as mean ± standard deviation. One‐way analysis of variance (ANOVA), two‐way ANOVA, and *t*‐tests were used. The results are categorized as follows: NS indicates no significance; ∗ denotes *p* < 0.05; ∗∗ denotes *p* < 0.01; and ∗∗∗ denotes *p* < 0.001.

## Author Contributions

The manuscript was written through contributions from all authors. All authors approved the final version of the manuscript. **Yifei Jiang**: Conceptualization, methodology, data curation, funding acquisition, formal analysis, writing – original draft, writing – review and editing; **Huizhen Yang**: Investigation, methodology, validation; **Guoping Jia**: Methodology, validation; **Wangxi Hai**: Investigation, validation; **Rongyi Huang**: Investigation, methodology; **Peng Wang**: Investigation, methodology; **Min Zhang**: Investigation, methodology; **Danni Li**: Investigation, methodology; **Yizhou Chen**: Investigation, methodology; **Xiao Li**: Investigation, supervision methodology, project administration; **Biao Li**: Investigation, supervision methodology, project administration; **Chunfu Zhang**: Conceptualization, supervision, funding acquisition, project administration, writing – review and editing.

## Ethics Statement

The animal experiments had the approval of the Animal Protection and Care Committee of Shanghai Jiao Tong University (Ethics No. 2023002).

## Conflicts of Interest

The authors declare no conflicts of interest.

## Supporting information




**Supporting File 1**: exp270144‐sup‐0001‐SuppMat.docx.

## Data Availability

The data that support the findings of this study are available from the corresponding author upon reasonable request.
