# Supplementary material for: Ultrasmall Au10–12 Nanoclusters: A Novel Platform for 223Ra‐Targeted Tumor α‐Therapy
Source: Exploration (Beijing). 2026 Feb 24;6(2):70144. doi: 10.1002/exp2.70144 (PMC13094526; doi:10.1002/exp2.70144)
Supplement: Supplementary file 1 — Supporting File 1: exp270144‐sup‐0001‐SuppMat.docx. [file EXP2-6-70144-s001.docx]

**Supporting Information**

**Ultrasmall Au_10-12_ Nanoclusters: a Novel Platform for ^223^Ra-targeted Tumor α-Therapy**

**1. Supporting Figures**


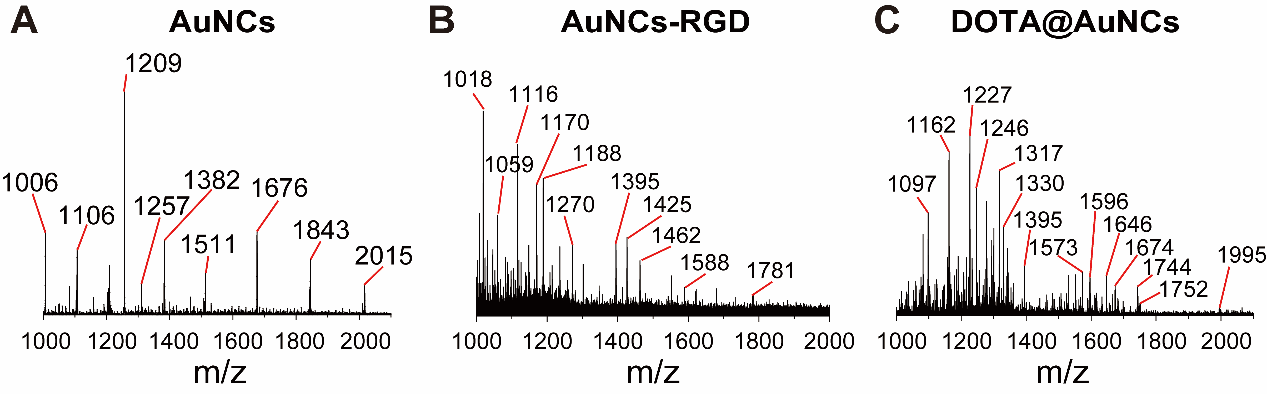


**Figure S1** ESI mass spectrum of AuNCs (A), RGD-functionalized AuNCs (AuNCs-RGD) (B) and DOTA@AuNCs (C).


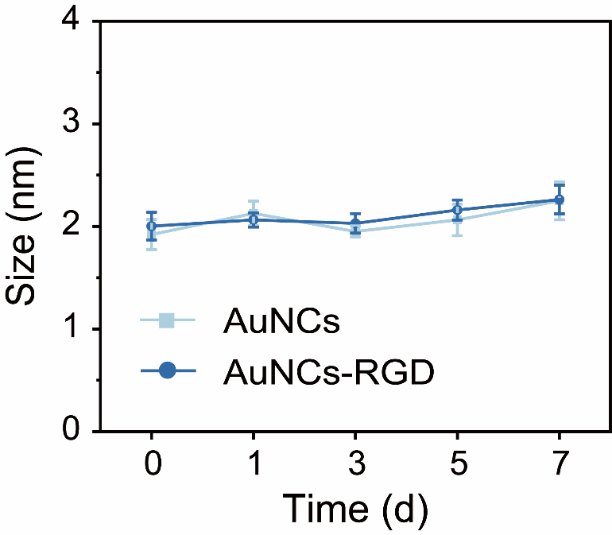


**Figure S2** The colloidal stability of AuNCs and AuNCs-RGD monitored by DLS. The clusters were dispersed in mouse serum.


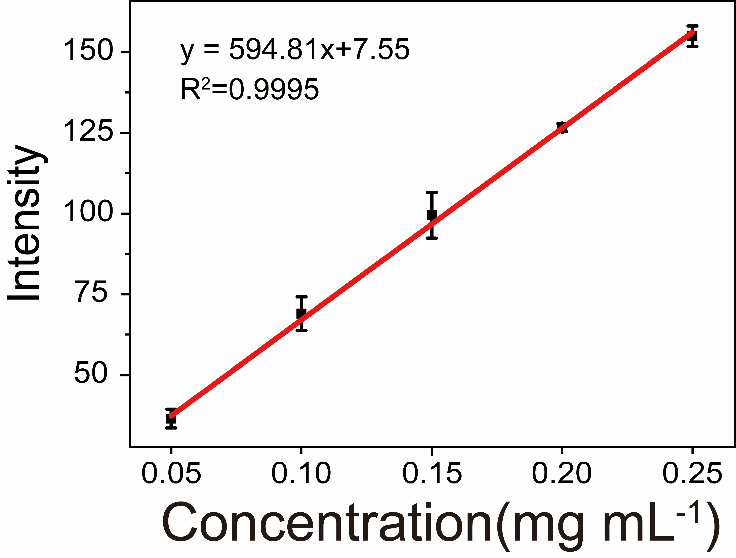


**Figure S3** Standard curve of fluorescent amine.





**Figure S4** Representative TEM image of DOTA@AuNCs.


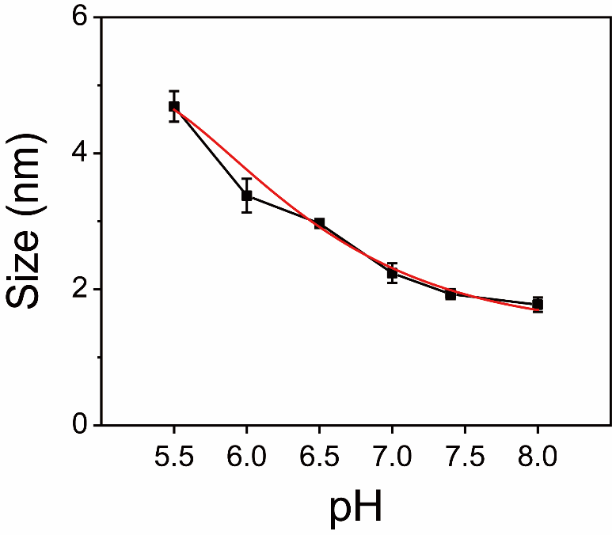


**Figure S5**. Size dependence of DOTA-AuNCs-RGD with pH measured by DLS.


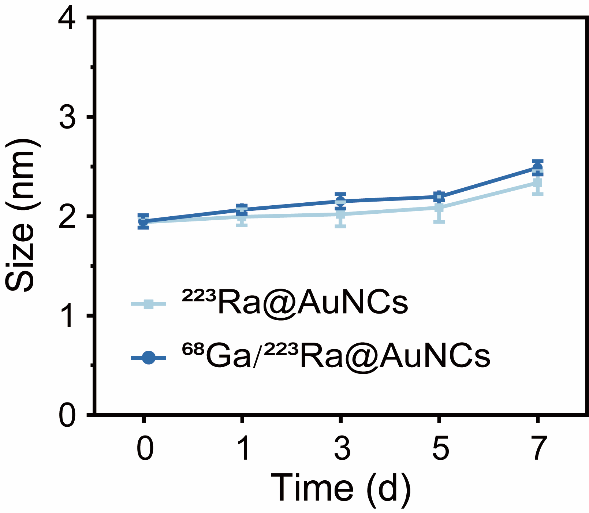


**Figure S6** The colloidal stability of ^223^Ra and ^223^Ra, ^68^Ga co-labeled AuNCs monitored by DLS. The clusters were dispersed in mouse serum.


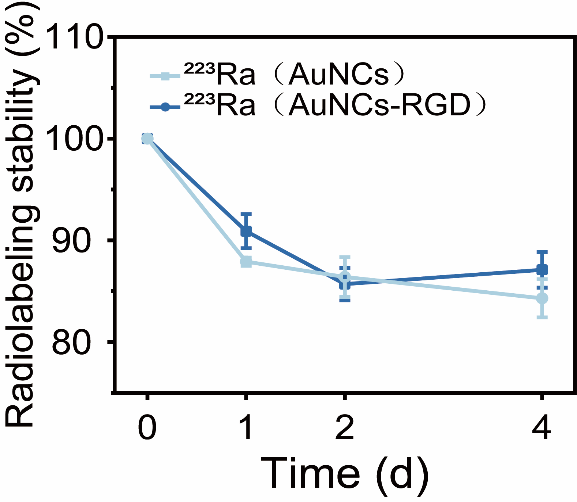


**Figure S7** Radiolabeling stability of ^223^Ra in ^223^Ra and ^68^Ga co-labeled AuNCs (^68^Ga/^223^Ra@AuNCs-RGD) within 4 days.


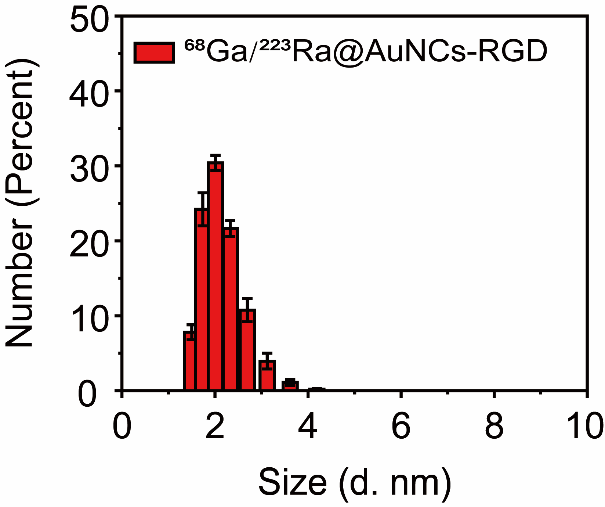


**Figure S8** The hydrodynamic size of ^68^Ga/^223^Ra@AuNCs-RGD determined by DLS.


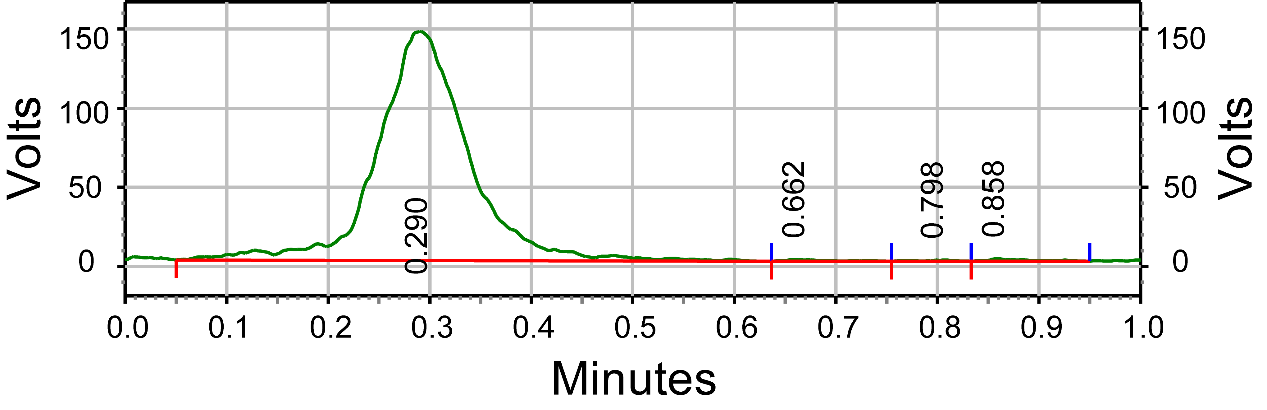


**Figure S9** Radio-TLC of ^68^Ga in ^68^Ga/^223^Ra@AuNCs-RGD after being dispersed in mouse serum for 30 min.


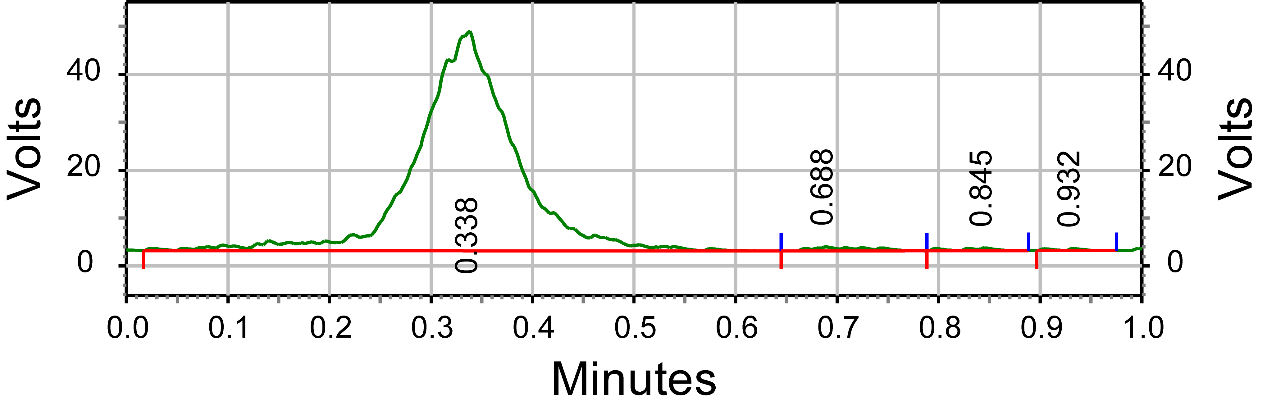


**Figure S10** Radio-TLC of ^68^Ga in ^68^Ga/^223^Ra@AuNCs-RGD after being dispersed in mouse serum for 60 min.


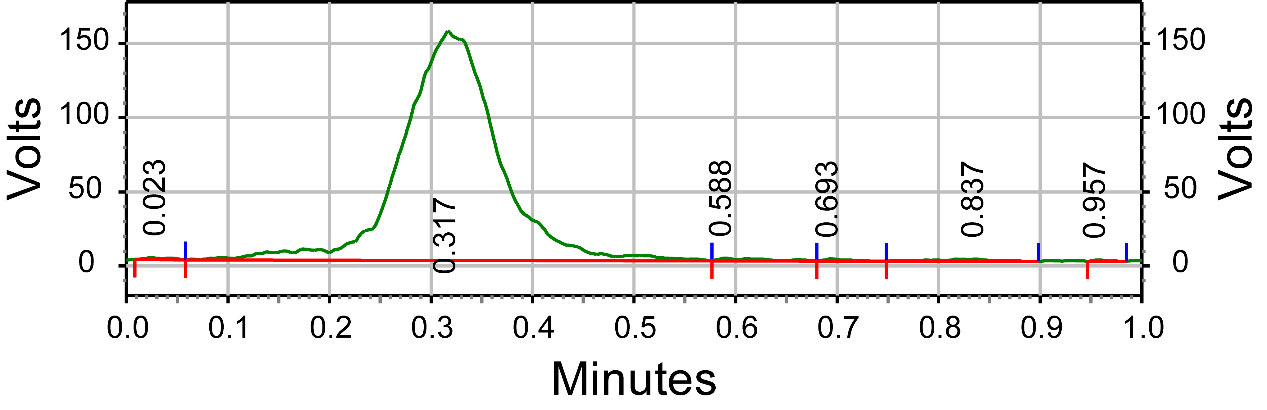


**Figure S11** Radio-TLC of ^68^Ga in ^68^Ga/^223^Ra@AuNCs-RGD after being dispersed in mouse serum for 120 min.


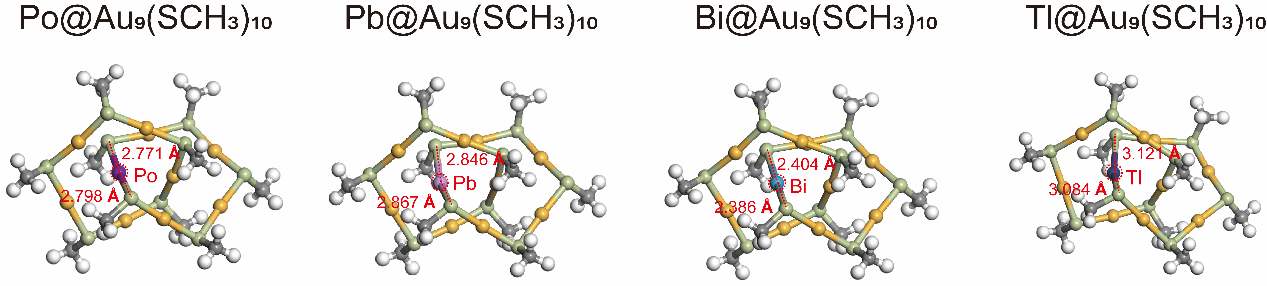


**Figure S12** The optimized structure of Po/Pb/Bi/Tl@Au_9_ clusters using ball-and-stick model and the bond length of Po, Pb, Bi and Tl atom with adjacent S atom was annotated. C atoms gray, H atoms white, S atoms light green, Au atoms yellow, Po atom dark violet, Pb atom light violet, Bi atom light blue and Tl atom dark blue.


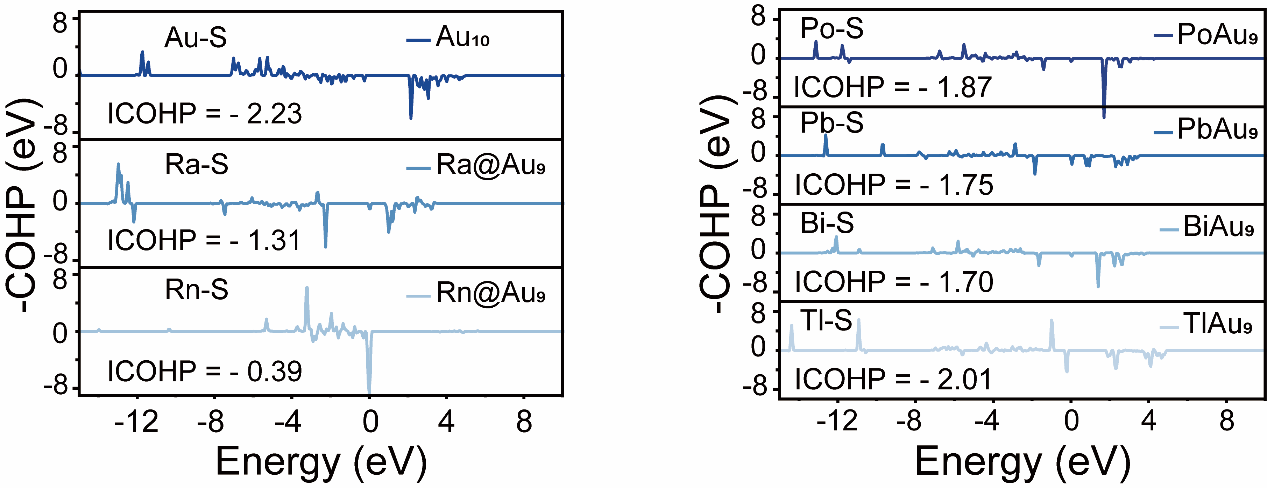


**Figure S13** Crystal orbital Hamilton population (COHP) analysis of the interactions between heteroatom and neighboring sulfur atom in the optimized structure of the heteroatom-doped Au_10_ clusters.


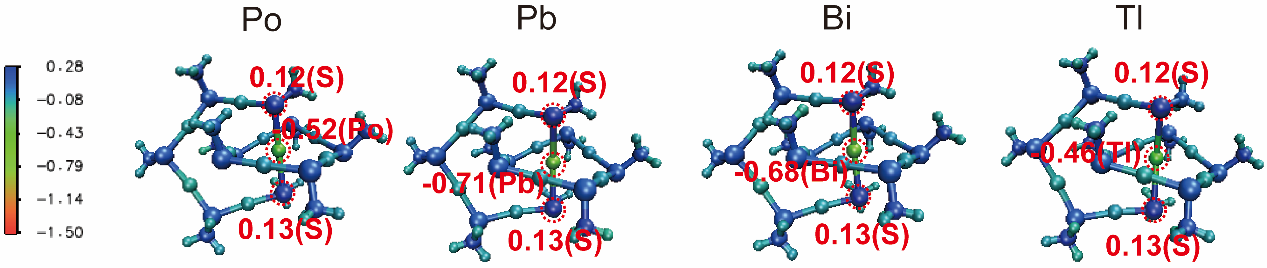


**Figure S14** Bader charge analyses of the Po/Pb/Bi/Tl-doped Au_10_ clusters. The numerical value indicates charge transfer of Po, Pb, Bi, Tl atom and adjacent S atoms.


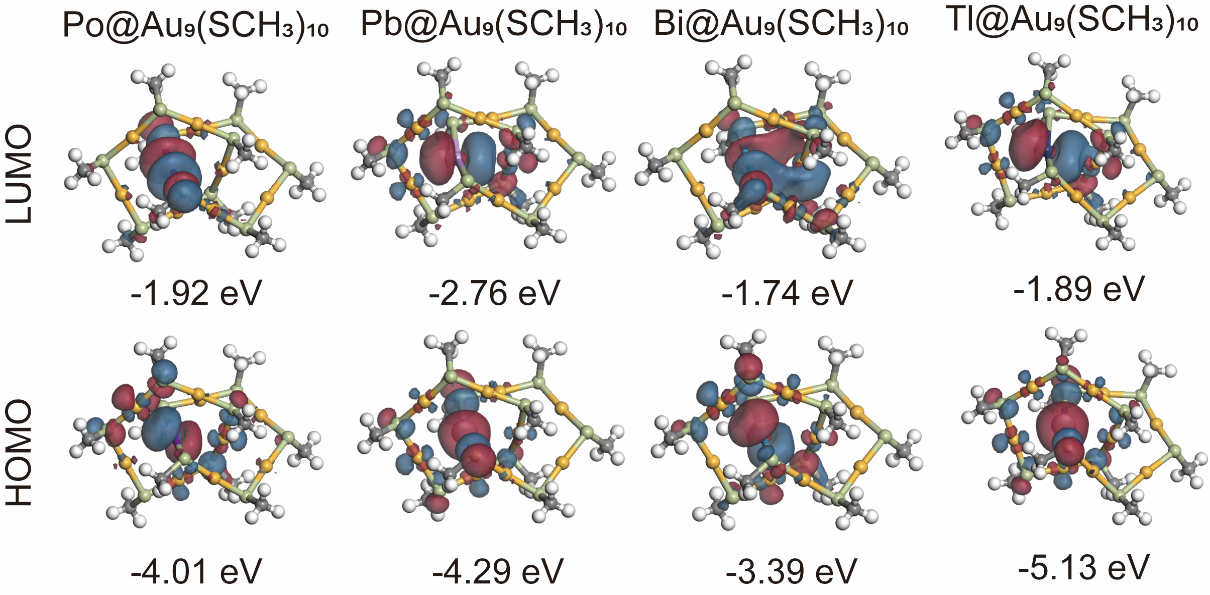


**Figure** **S15** HOMO−LUMO gaps for the Po/Pb/Bi/Tl-doped Au_10_ clusters and their corresponding energy levels.


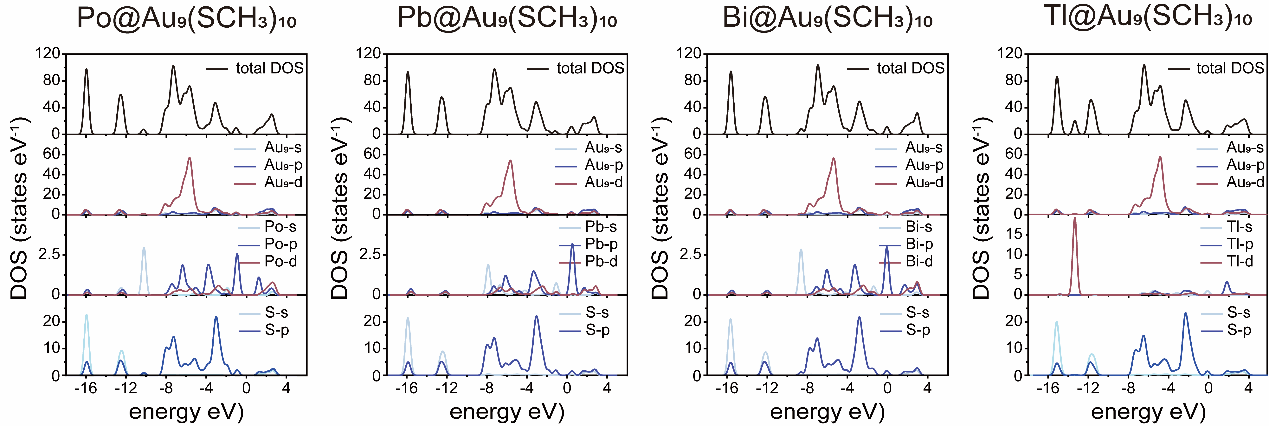


**Figure S16** Total and partial density of states for the Po/Pb/Bi/Tl-doped Au_10_ clusters. The Fermi level is presented as a dashed vertical line and shifted to zero.


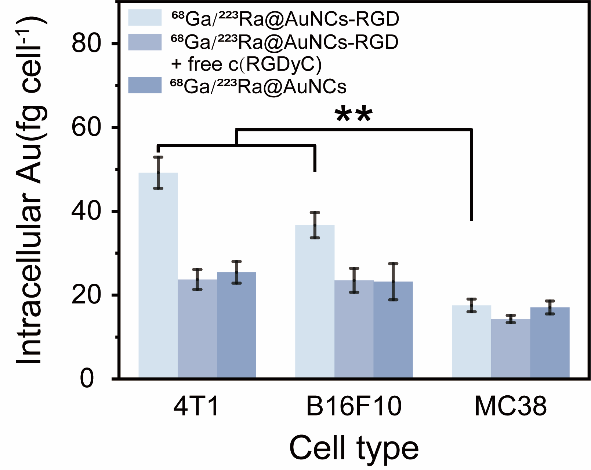


**Figure S17** *In vitro* cellular uptake assay of the AuNCs (after complete decay of ^223^Ra and ^68^Ga) in diverse cancer cell lines. The intracellular gold contents were quantified by ICP-MS.


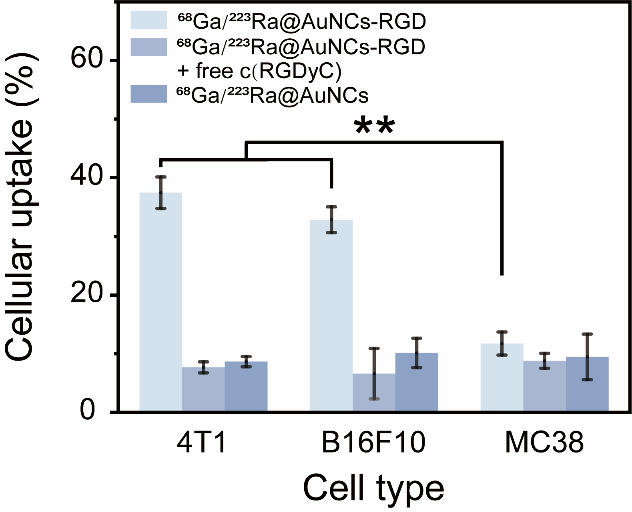


**Figure S18** Cellular uptake of ^68^Ga-labeled ^223^Ra@AuNCs-RGD (after complete decay of ^223^Ra). The intracellular radioactivity of ^68^Ga was measured by a gamma counter.


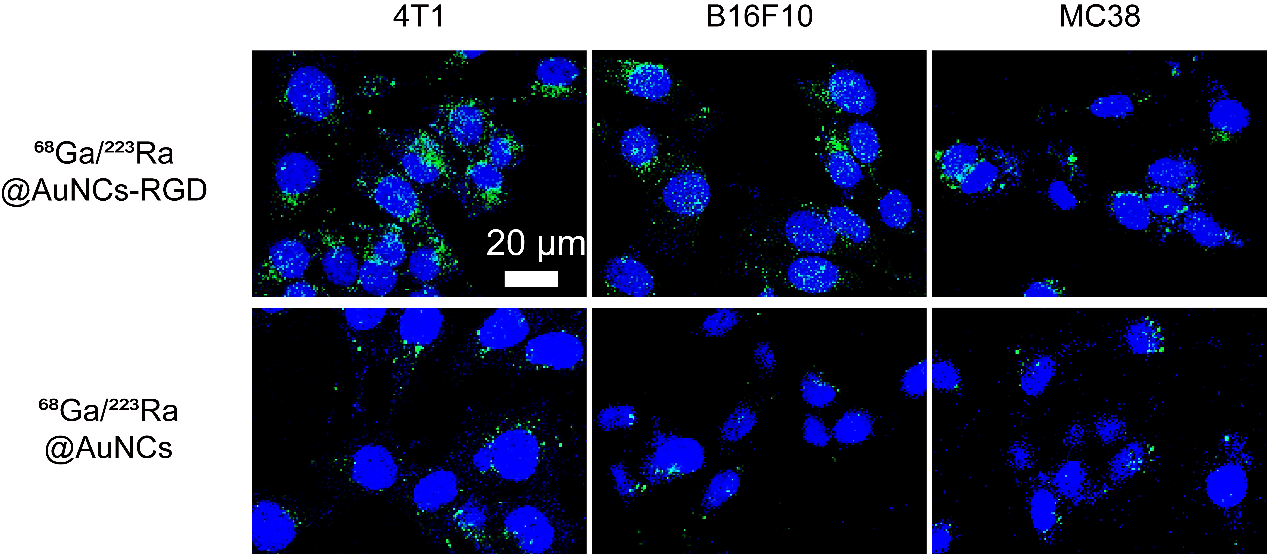


**Figure S19** Confocal immunofluorescence photomicrographs of cells treated with FITC-tagged ^68^Ga/^223^Ra@AuNCs or ^68^Ga/^223^Ra@AuNCs-RGD after complete decay of ^223^Ra and ^68^Ga. Cell nuclei were stained with DAPI (blue). Scale bar = 20 µm.


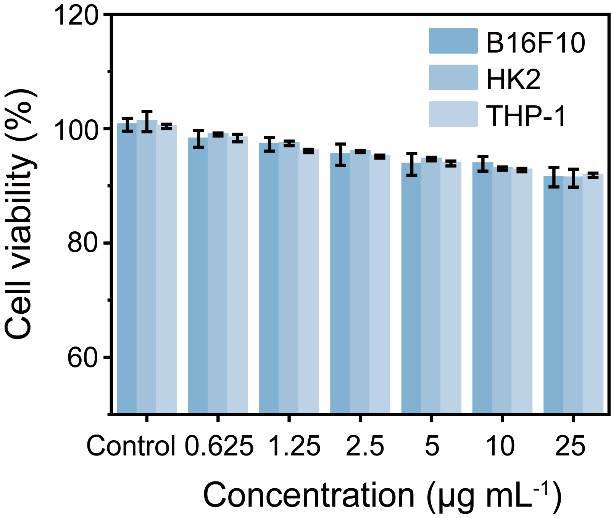


**Figure S20** Cytotoxicity of ^68^Ga/^223^Ra@AuNCs-RGD (after complete decay of ^223^Ra and ^68^Ga) on tumor cells (B16F10) and normal cells (HK-2 and THP-1) after incubation for 24 h.


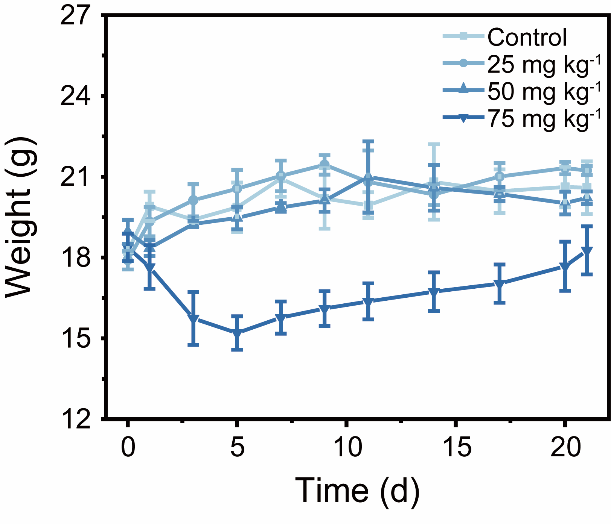


**Figure S21** Body weights of mice treated with different doses of ^68^Ga/^223^Ra@AuNCs-RGD after ^68^Ga/^223^Ra complete decay within 12 days.


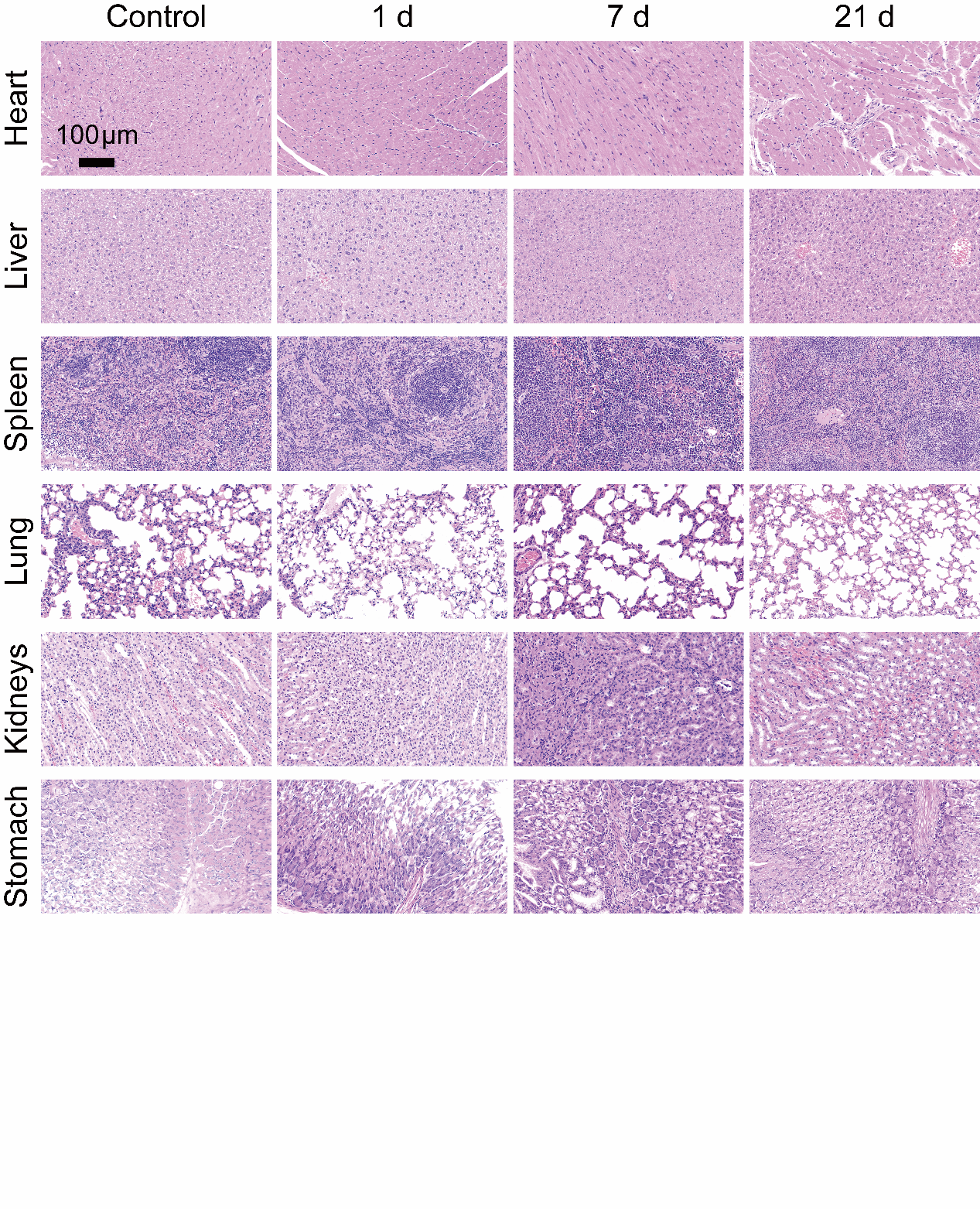


**Figure S22** HE staining of the heart, liver, spleen, lung, kidney and stomach from mice following treatment with ^68^Ga/^223^Ra@AuNCs-RGD after ^68^Ga/^223^Ra completely decayed at the dose of 50 mg Au kg^-1^ for 1, 7, and 21 days*.*


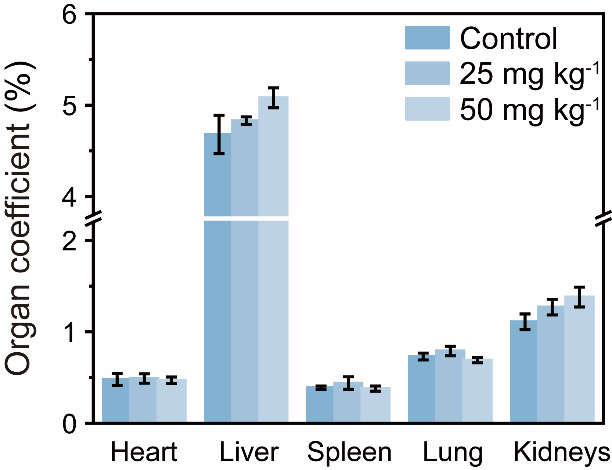


**Figure S23** The organ coefficients of mice treated with^68^Ga/^223^Ra@AuNCs-RGD after ^68^Ga/^223^Ra complete decay for 21 days.


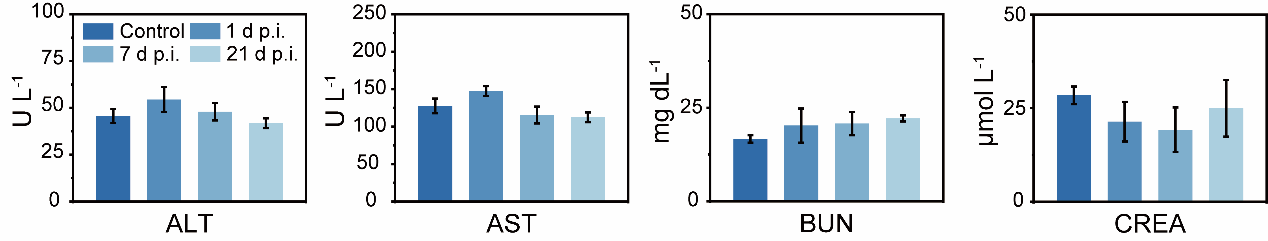


**Figure S24** Biochemical levels of the liver function (ALT and AST) and the kidney function (BUN and CREA) when the mice were treated with ^68^Ga/^223^Ra@AuNCs-RGD after ^68^Ga/^223^Ra completely decayed at the dose of 50 mg Au kg^-1^ b.w. for 1, 7, and 21 days *p.i.*


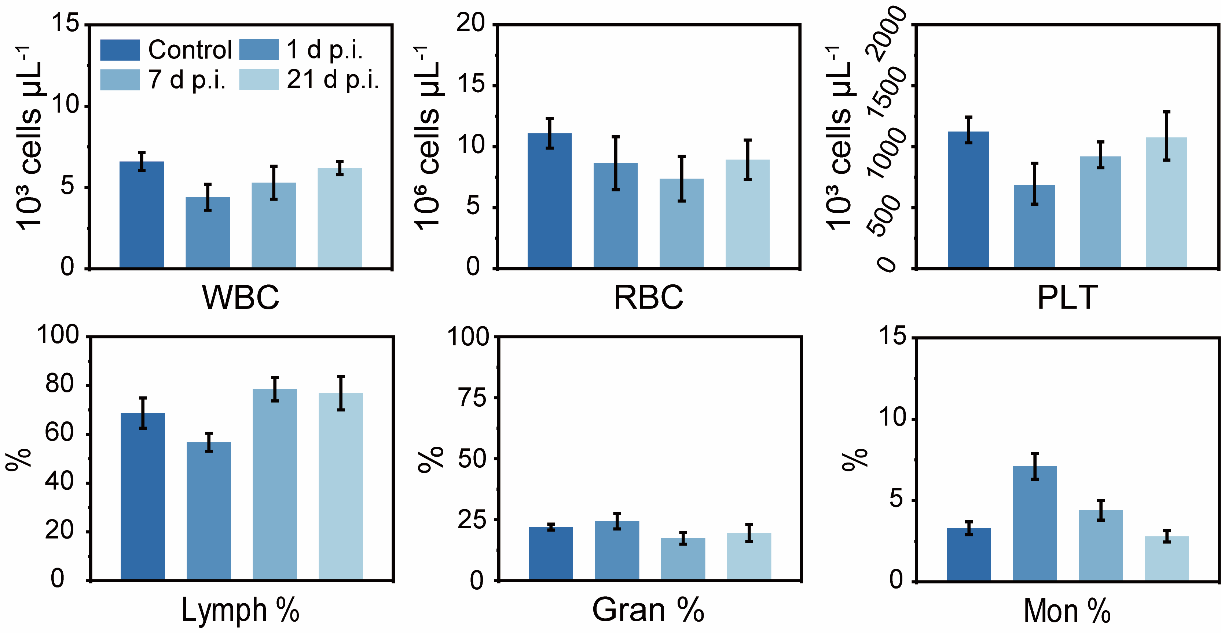


**Figure S25** Hematology of WBC, RBC, PLT, Lymph%, Gran% and Mon% when the mice were treated with ^68^Ga/^223^Ra@AuNCs-RGD after ^68^Ga/^223^Ra completely decayed at the dose of 50 mg Au kg^-1^ b.w. for 1, 7, and 21 days *p.i.*.


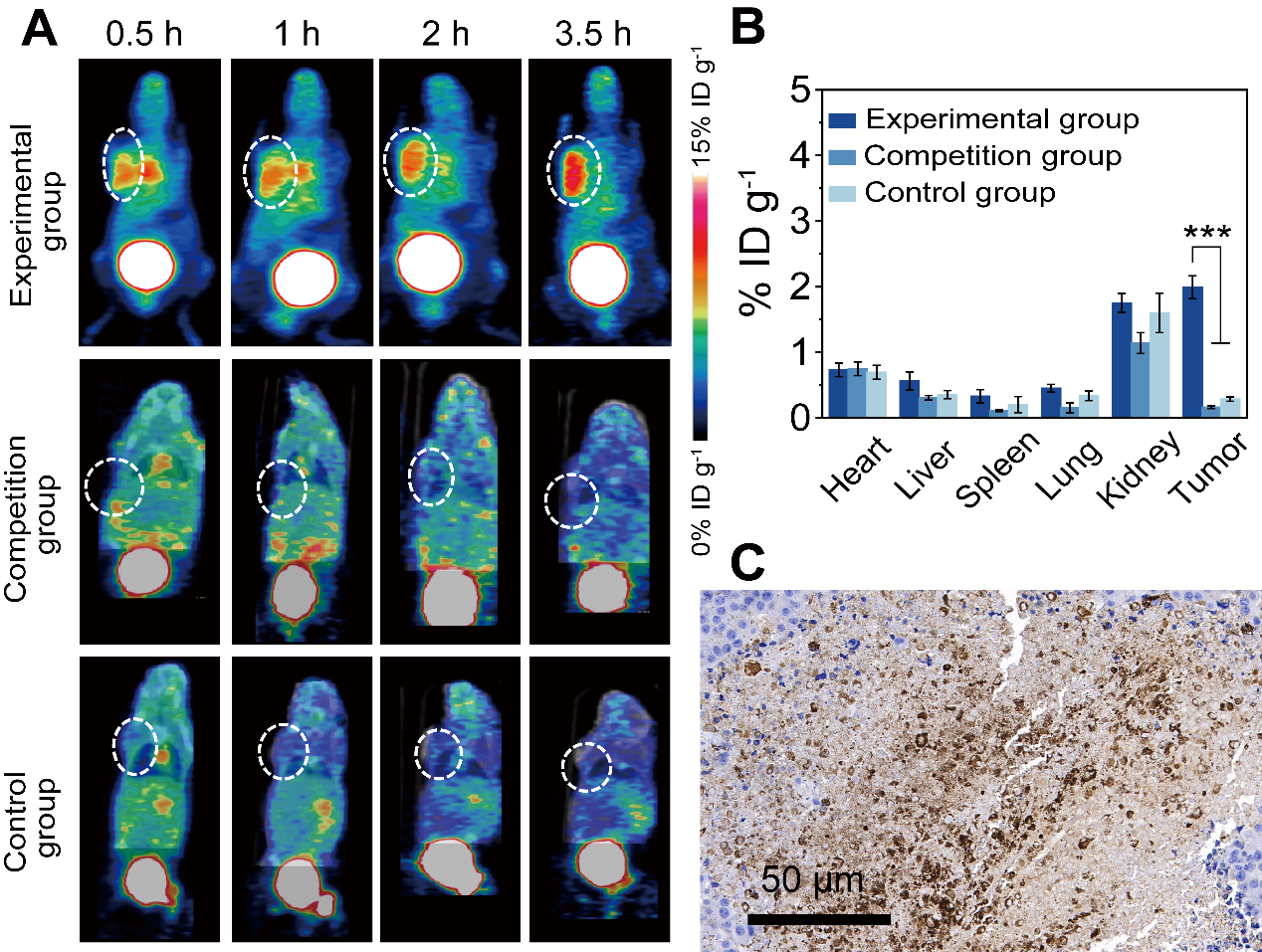


**Figure S26** Tumor targeting capability of ^68^Ga/^223^Ra@AuNCs-RGD. (A) PET/CT imaging of the B16F10 tumor bearing mice after *i.v* injection of ^68^Ga/^223^Ra@AuNCs-RGD (experimental group), ^68^Ga/^223^Ra@AuNCs-RGD plus free RGD (competition group) and ^68^Ga/^223^Ra@AuNCs (control group) (12.5 mCi kg^-1^ for ^68^Ga, 50 mg Au kg^-1^ b.w.).The white circle denotes the tumor site. (B) Biodistribution of different probes in B16F10 tumor bearing mice at 3.5 h p.i; (C) α_v_β_3_ staining of representative tumor tissue from B16F10 mice. The data represents the mean ± s. d., n = 3 biologically independent mice. The error bars represent s.d. values. Analyzed by two-way ANOVA, followed by Bonferroni’s multiple comparisons test.


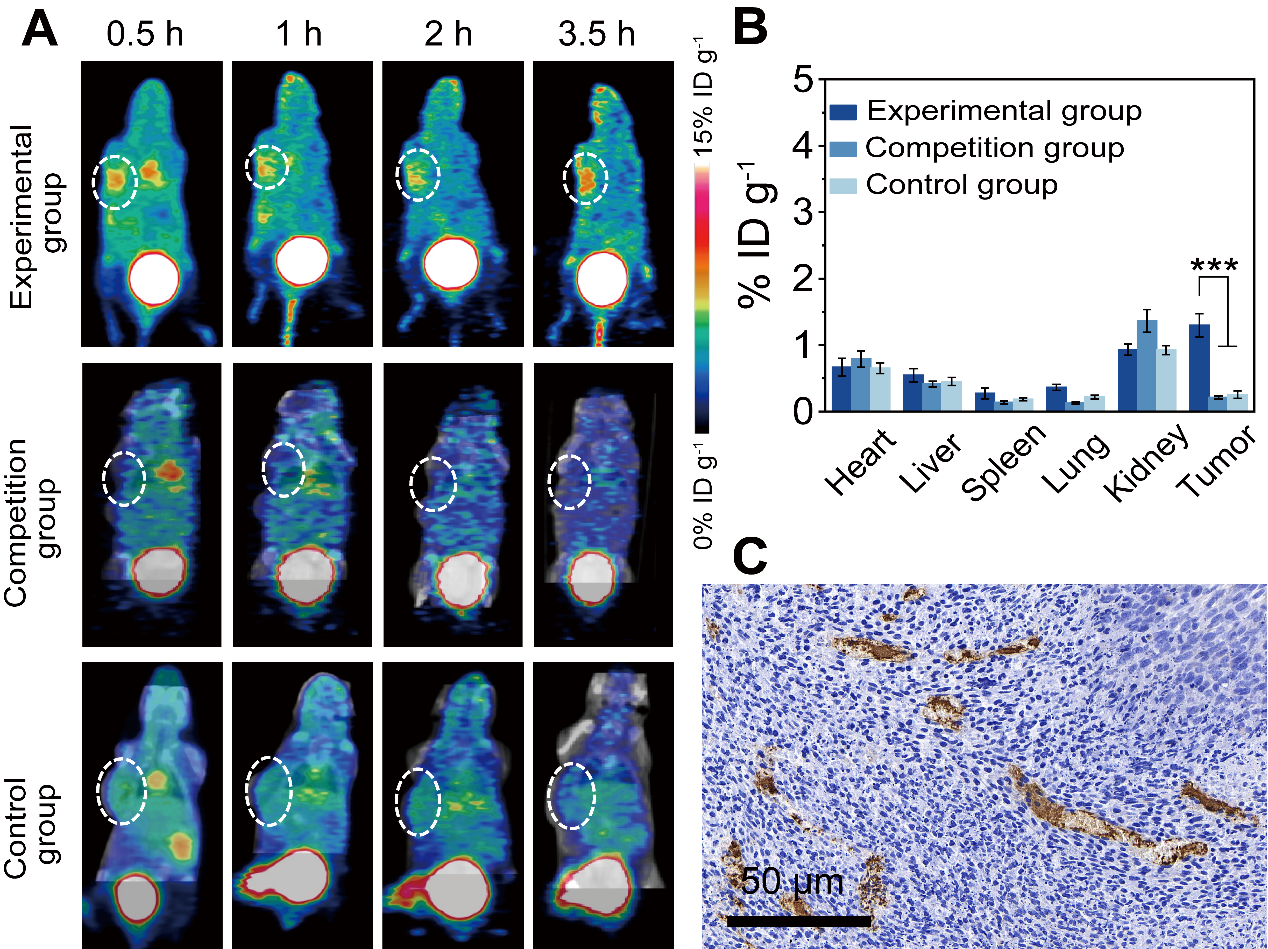


**Figure S27** Tumor targeting capability of ^68^Ga/^223^Ra@AuNCs-RGD. (A) PET/CT imaging of the MC38 tumor bearing mice after *i.v* injection of ^68^Ga/^223^Ra@AuNCs-RGD (experimental group), ^68^Ga/^223^Ra@AuNCs-RGD plus free RGD (competition group) and ^68^Ga/^223^Ra@AuNCs (control group) (12.5 mCi kg^-1^ for ^68^Ga, 50 mg Au kg^-1^ b.w.).The white circle denotes the tumor site. (B) Biodistribution of different probes in MC38 tumor bearing mice at 3.5 h p.i; (C) α_v_β_3_ staining of representative tissue from MC38 tumor. The data represents the mean ± s.d., n = 3 biologically independent mice. The error bars represent s.d. values. Analyzed by two-way ANOVA, followed by Bonferroni’s multiple comparisons test.


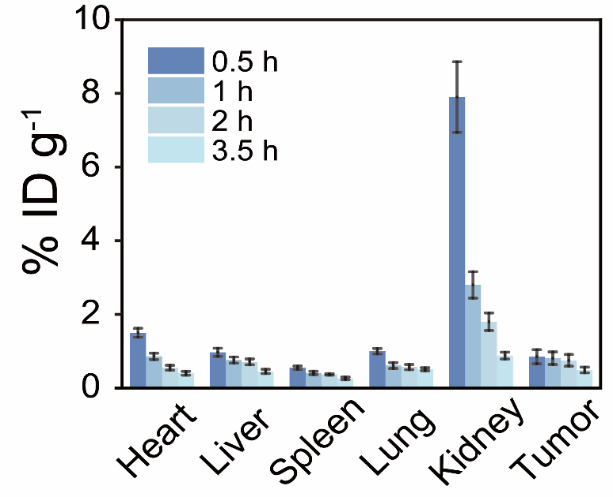


**Figure S28** Biodistribution of ^68^Ga/^223^Ra@AuNCs-RAD in 4T1 tumor bearing mice within 3.5 h.


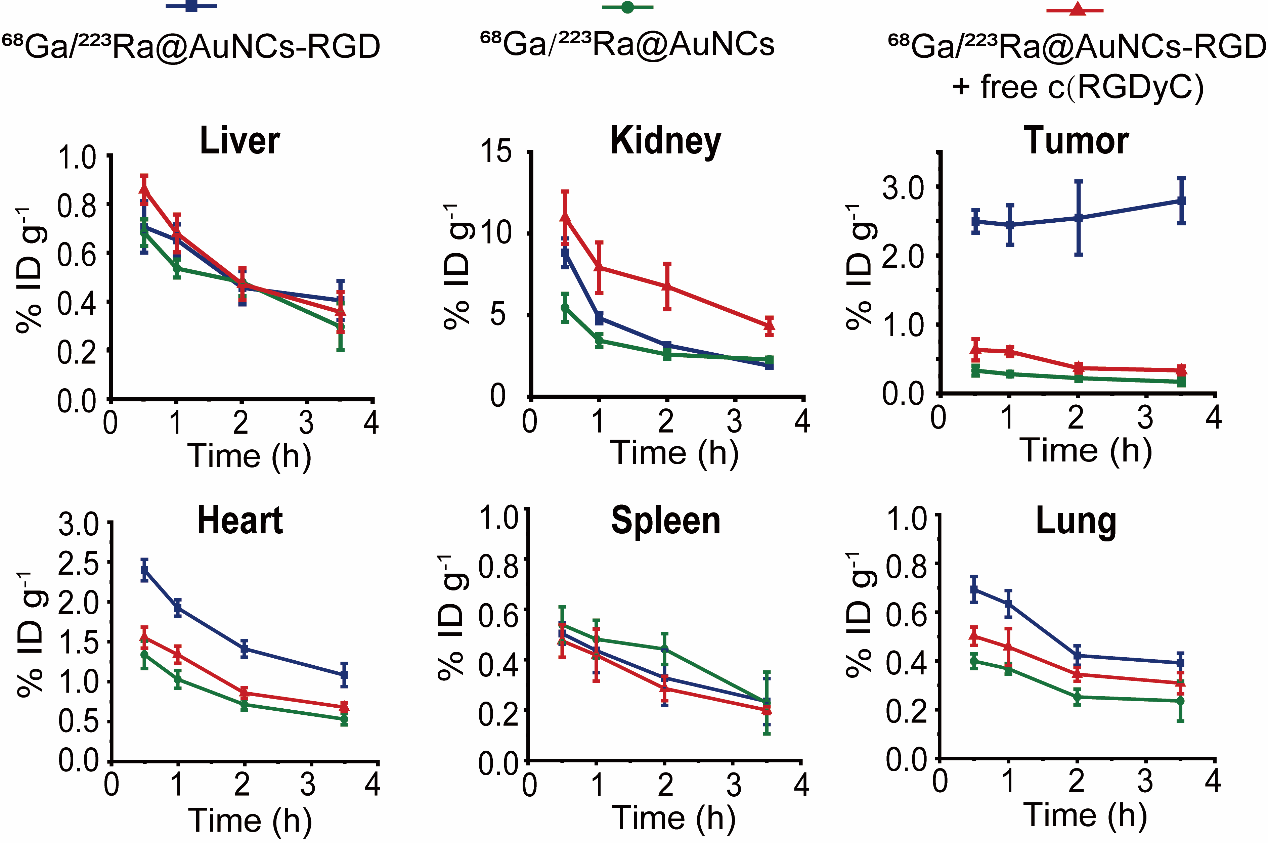


**Figure S29** ROI analyses of time-dependent signal intensity within 4T1 tumor bearing mice.


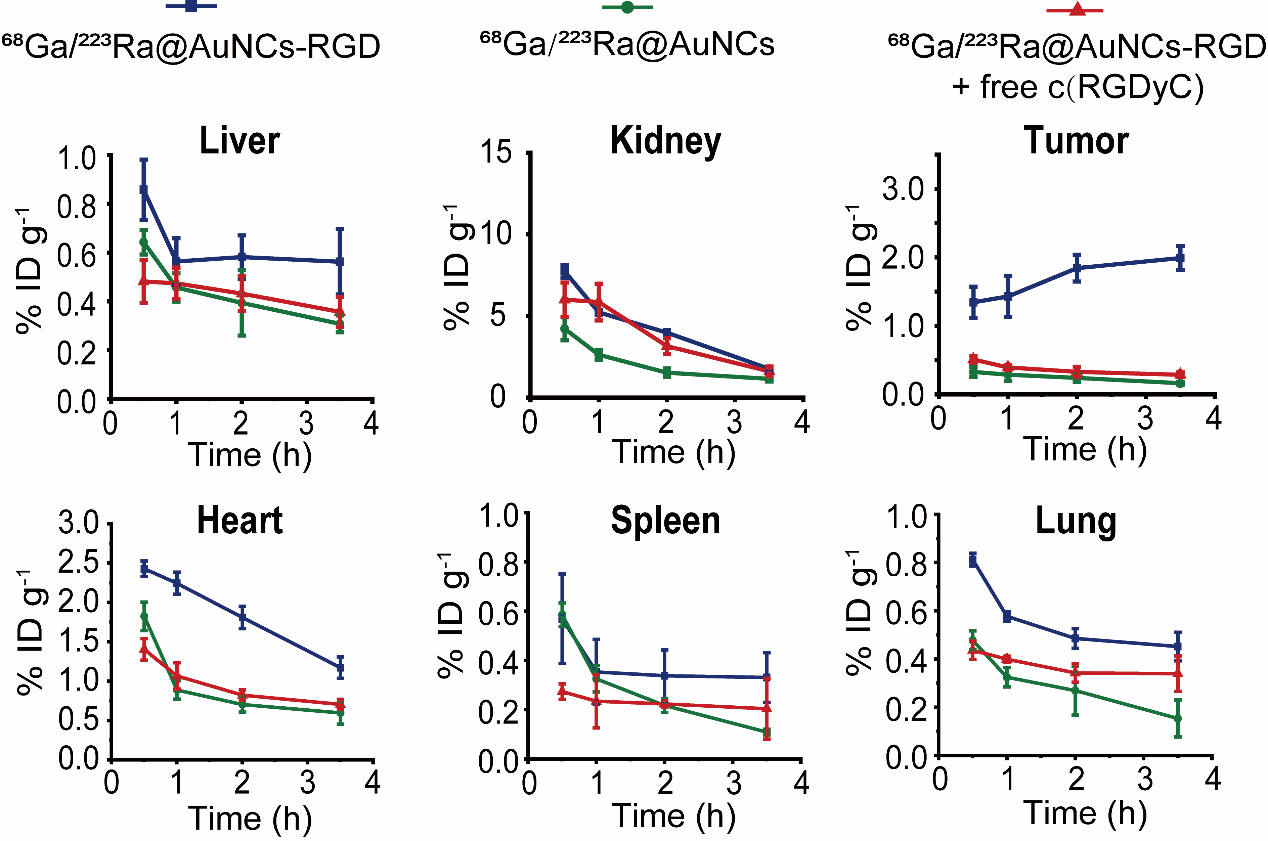


**Figure S30** ROI analyses of time-dependent signal intensity within B16F10 tumor bearing mice.


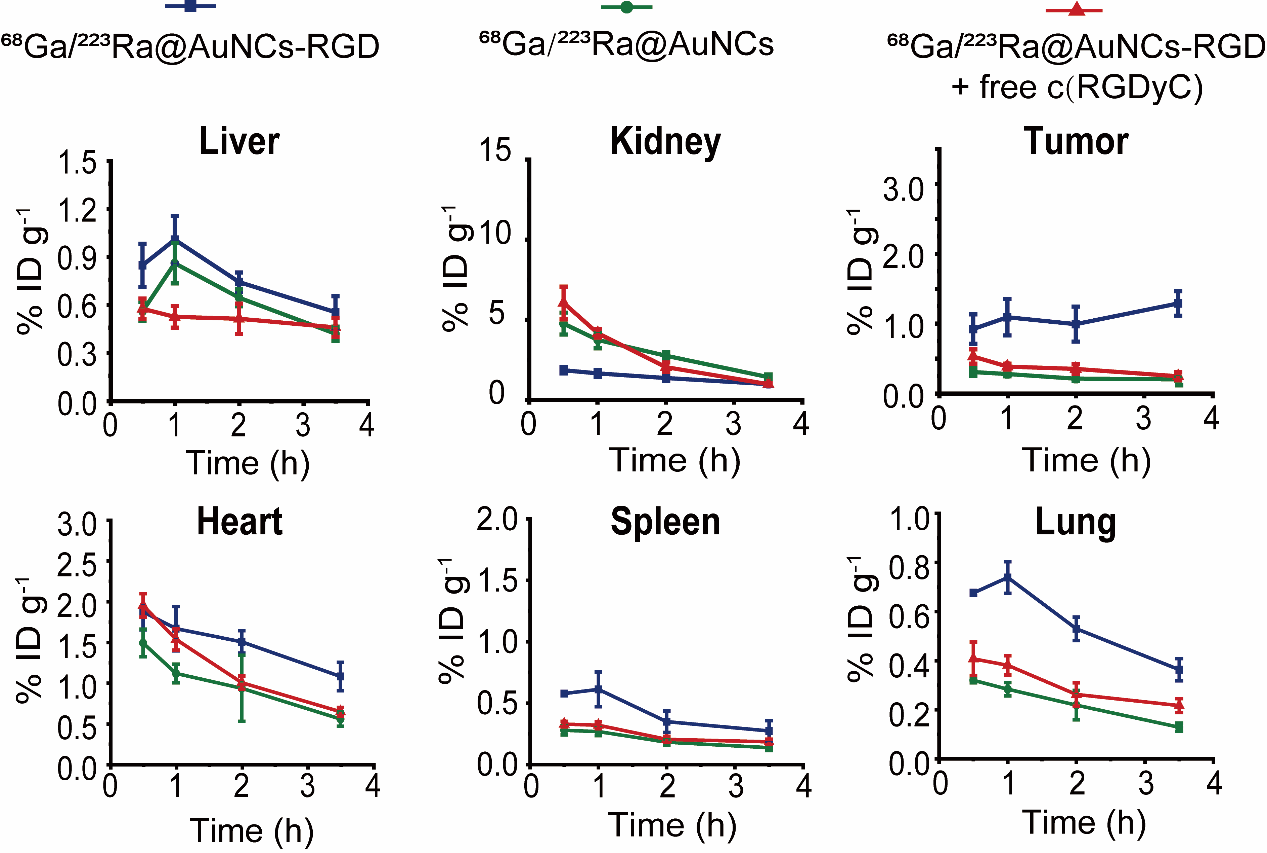


**Figure S31** ROI analyses of time-dependent signal intensity within MC38 tumor bearing mice.


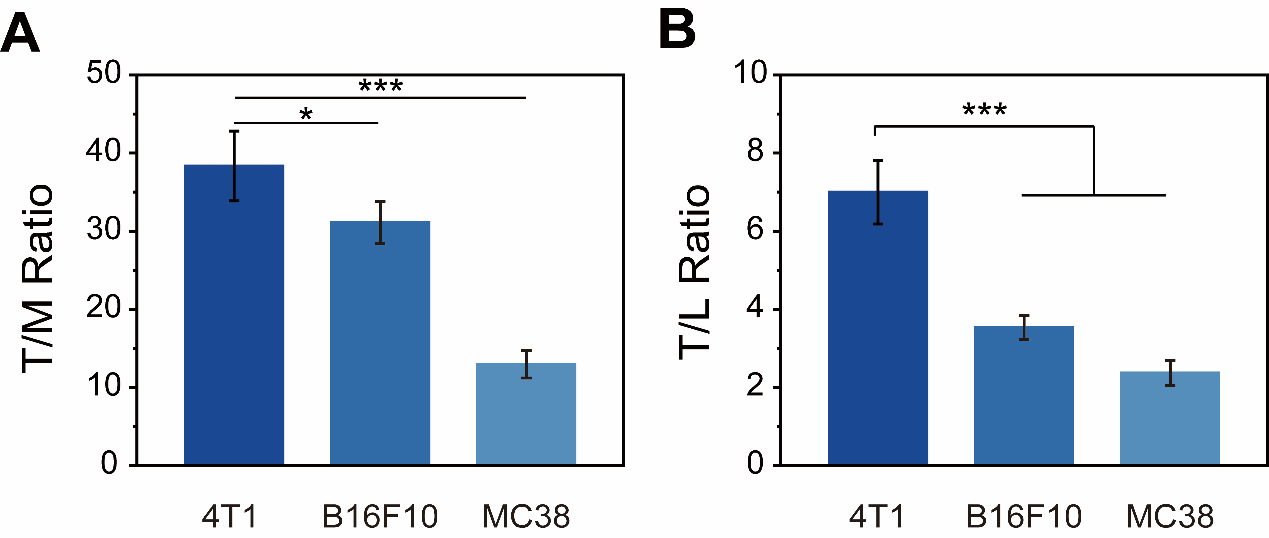


**Figure S32** Quantitative analysis of the tumor-to-muscle signal ratio(T/M) (A) and the tumor-to-liver signal ratio (T/L) (B) of ^68^Ga/^223^Ra@AuNCs-RGD at 3.5 h p.i.


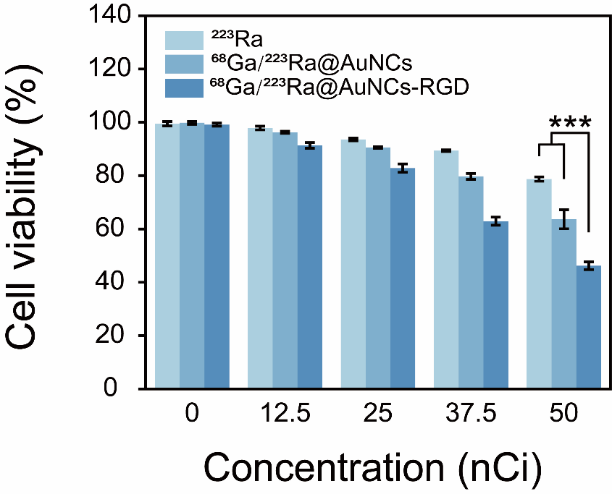


**Figure S33** Viability of 4T1 cells treated with free ^223^Ra, ^68^Ga/^223^Ra@AuNCs and ^68^Ga/^223^Ra@AuNCs-RGD at different doses of ^223^Ra after incubation for 24 h.


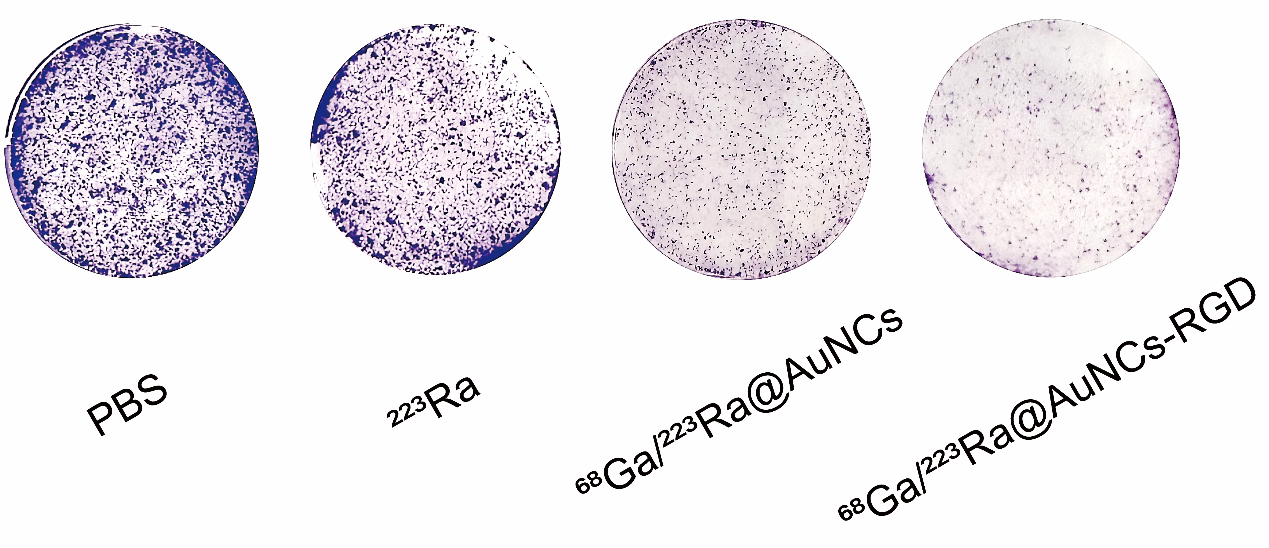


**Figure S34** Crystal violet staining of 4T1 cells treated with the PBS, free ^223^Ra, ^68^Ga/^223^Ra@AuNCs and ^68^Ga/^223^Ra@AuNCs-RGD for 24 h at a dosage of 50 nCi for ^223^Ra.


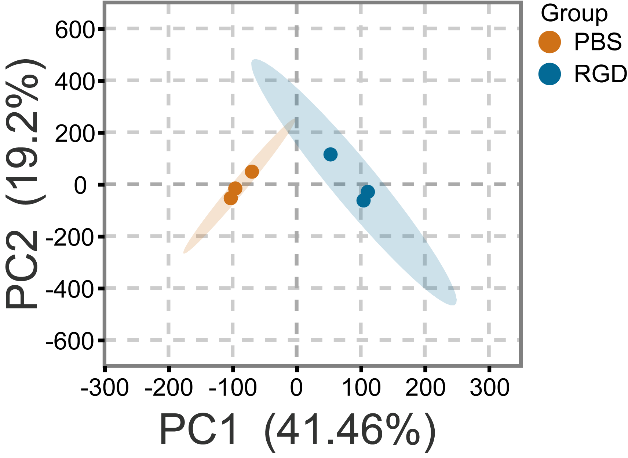


**Figure S35** PCA of proteins for different cell samples, indicative of protein expression differences between PBS and ^68^Ga/^223^Ra@AuNCs-RGD.


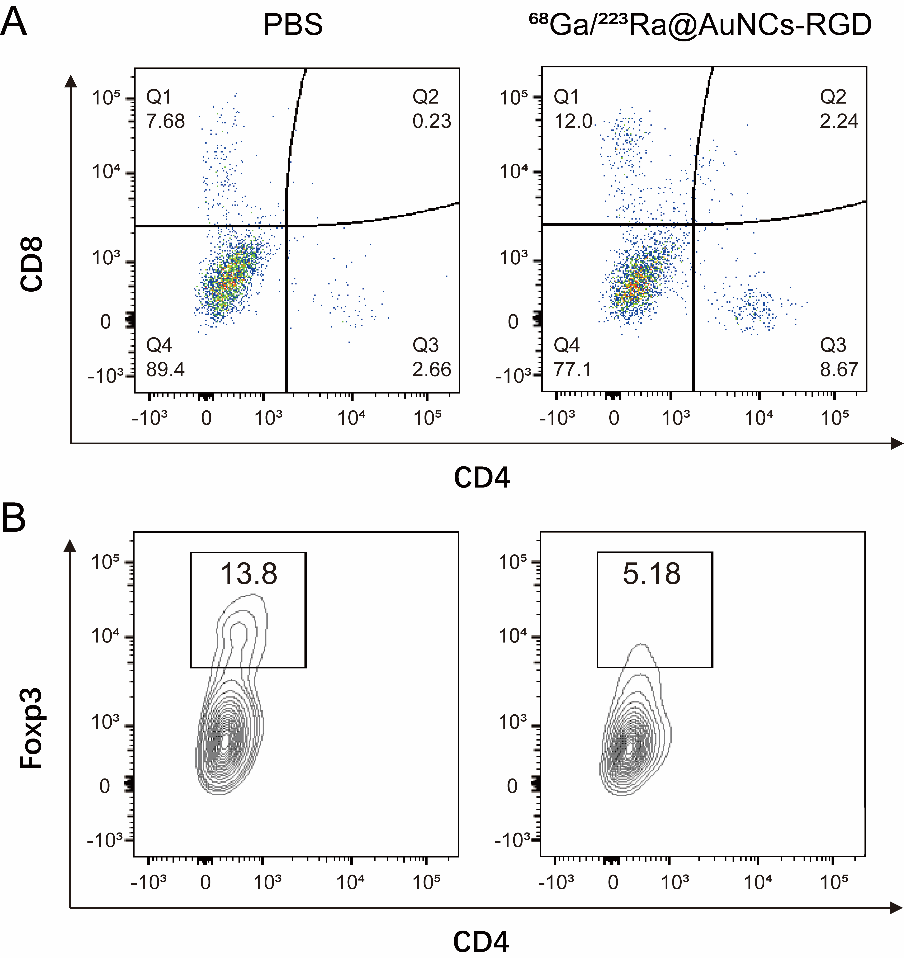


**Figure S36** Representative flow cytometry plots showing T cells and Treg cells in tumors from the PBS and ^68^Ga/^223^Ra@AuNCs-RGD groups after 7 days of treatment.

**
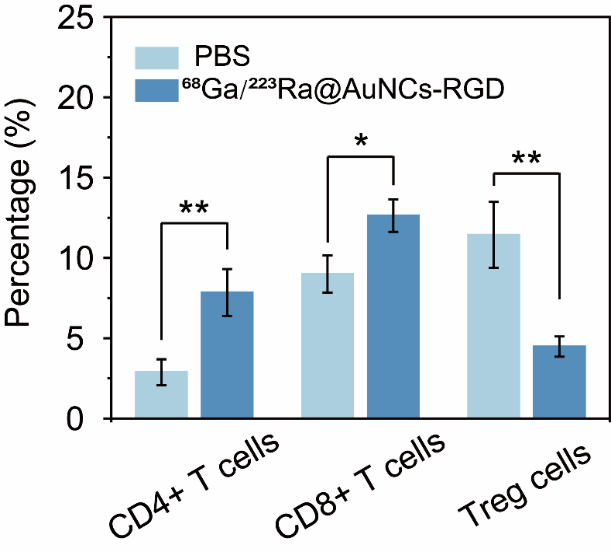
**

**Figure S37** Statistical data showing T cells and Treg cells in tumors from the PBS and ^68^Ga/^223^Ra@AuNCs-RGD groups after 7 days of treatment.


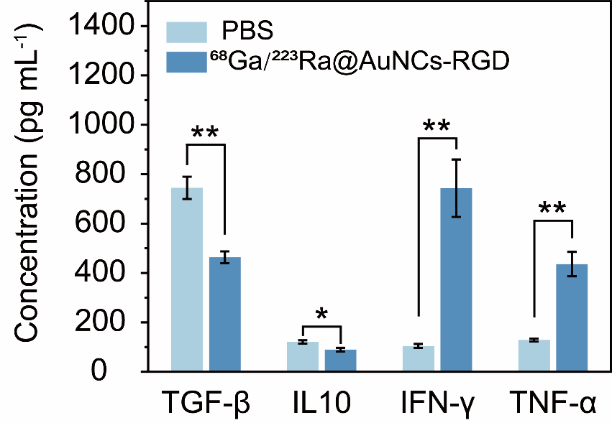


**Figure S38** Cytokine concentrations of TGF-β, IL-10, IFN-γ, and TNF-α. Data are presented as mean ± s.d.


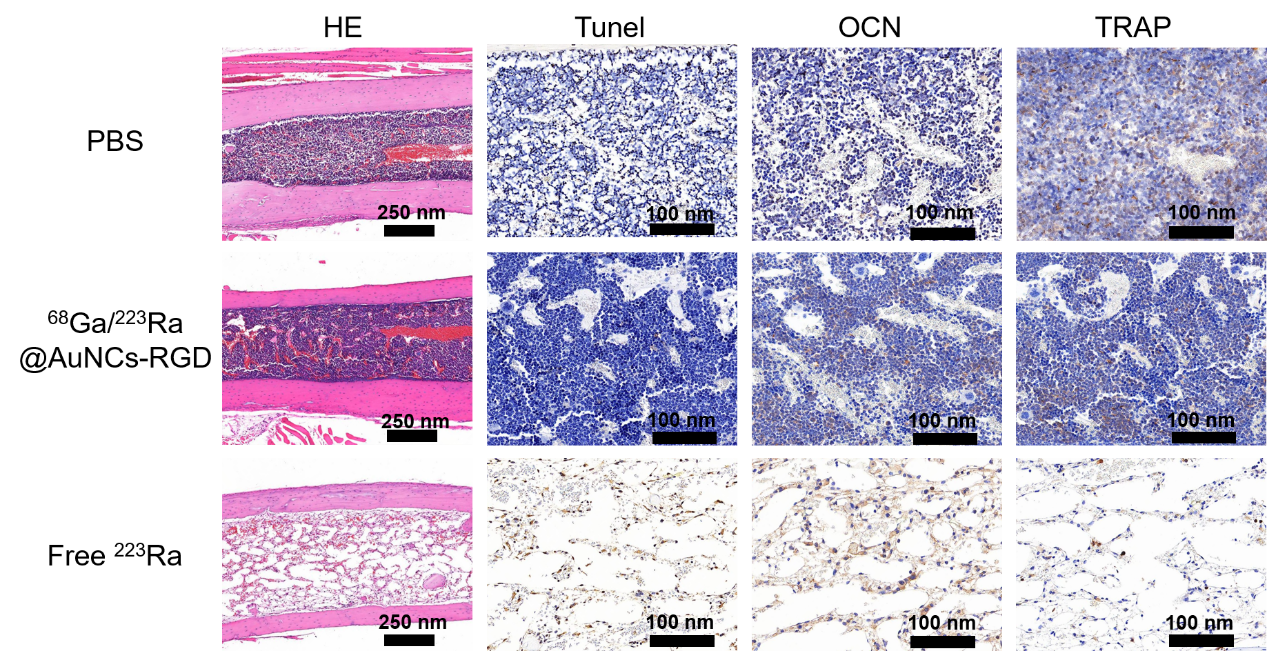


**Figure** **S39** Histological staining (DAPI/TUNEL, H&E, OCN and TRAP) of the leg bone tissues of the mice at the end of different treatments. Scale bar: 100 µm.

**
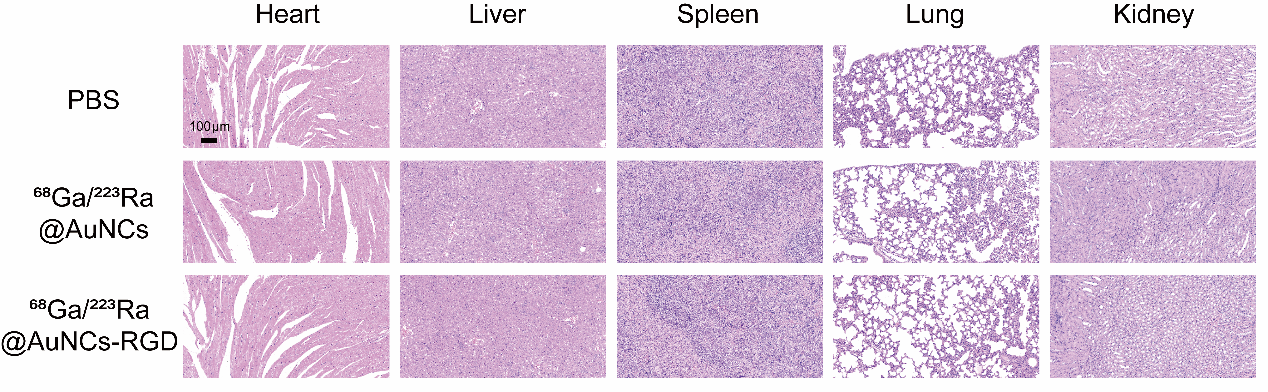
**

**Figure S40** H&E staining of major organs of the mice at the end of different treatments. Scale bar: 100 µm.


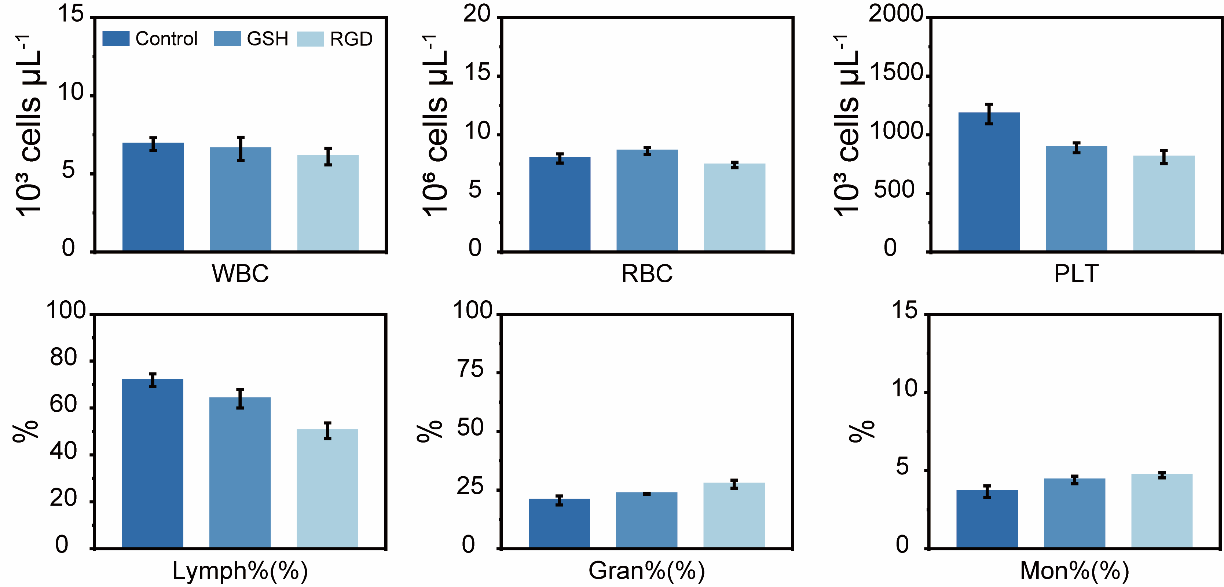


**Figure S41** Hematology of WBC, RBC, PLT, Lymph%, Gran% and Mon% at the end of different treatments. GSH was abbreviated of ^68^Ga/^223^Ra@AuNCs and RGD was abbreviated of ^68^Ga/^223^Ra@AuNCs-RGD.


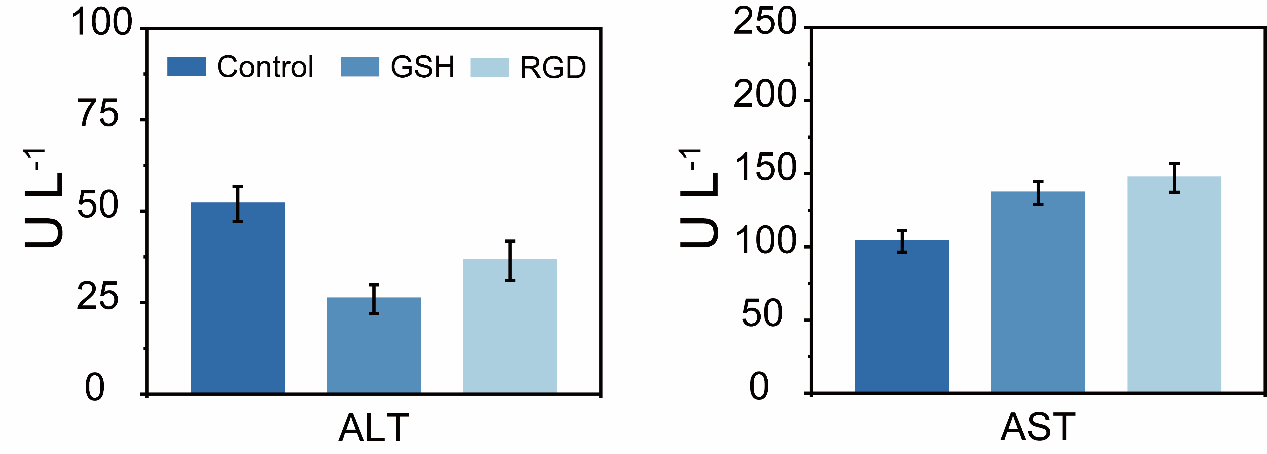


**Figure S42** Biochemical levels of the liver function (ALT and AST) at the end of different treatments. GSH was abbreviated of ^68^Ga/^223^Ra@AuNCs and RGD was abbreviated of ^68^Ga/^223^Ra@AuNCs-RGD.


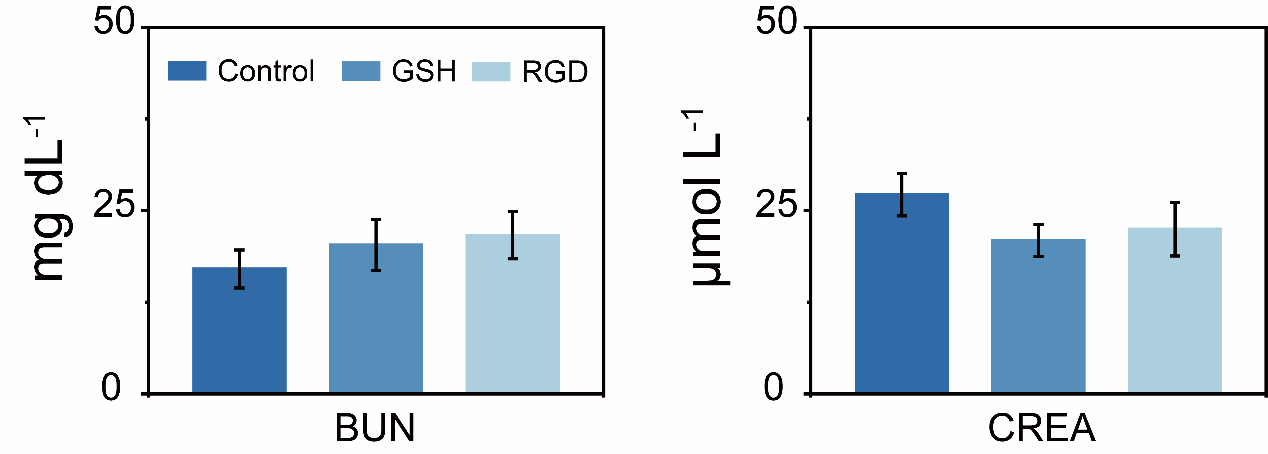


**Figure S43** Biochemical levels of the kidney function (BUN and CREA) at the end of different treatments. GSH was abbreviated of ^68^Ga/^223^Ra@AuNCs and RGD was abbreviated of ^68^Ga/^223^Ra@AuNCs-RGD.


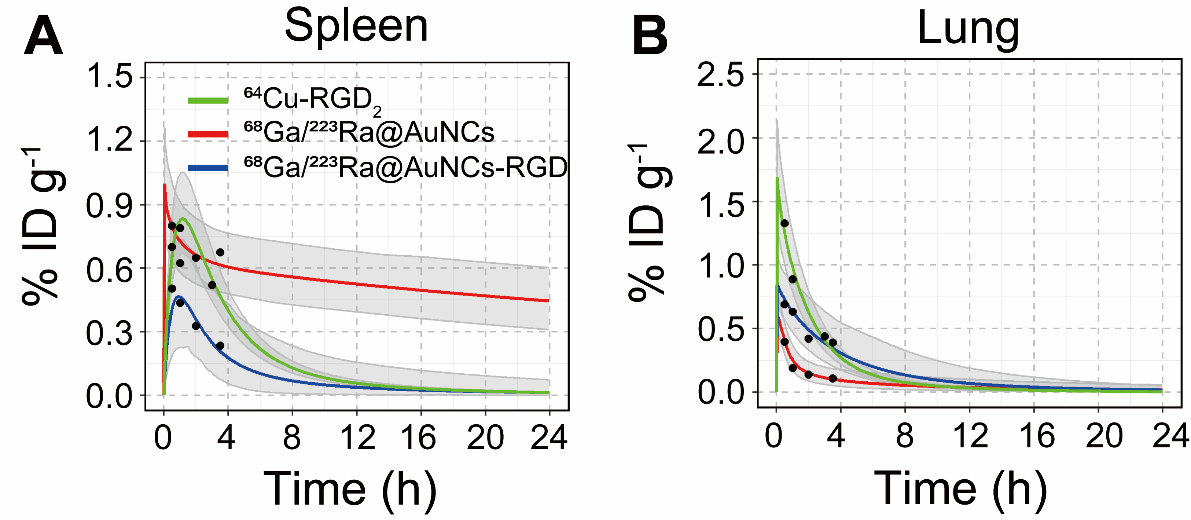


**Figu**r**e S44** The biodistribution of the ^68^Ga/^223^Ra-labeled AuNCs in spleen (A) and lung (B) (n = 3). The dots indicate the observed value, the curves indicate predicted means and the shaded area indicates the 95% prediction interval.


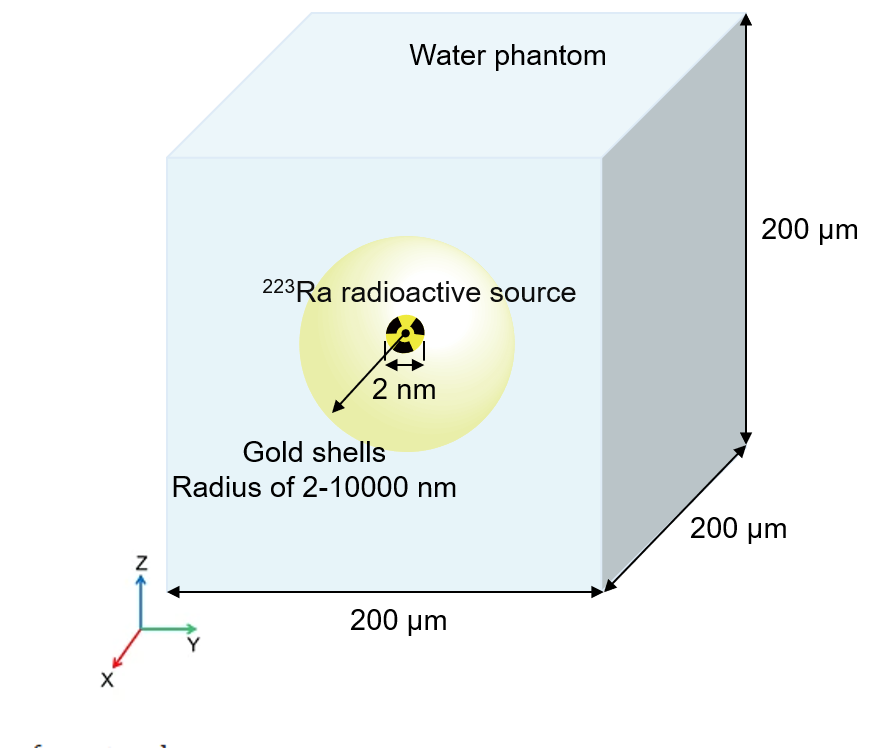


**Figu**r**e S45** Geometric setup for water phantom
